# Stability and sub-cellular localization of DNA polymerase β is regulated by interactions with NQO1 and XRCC1 in response to oxidative stress

**DOI:** 10.1093/nar/gkz293

**Published:** 2019-04-26

**Authors:** Qingming Fang, Joel Andrews, Nidhi Sharma, Anna Wilk, Jennifer Clark, Jana Slyskova, Christopher A Koczor, Hannes Lans, Aishwarya Prakash, Robert W Sobol

**Affiliations:** 1 University of South Alabama Mitchell Cancer Institute, 1660 Springhill Avenue, Mobile, AL 36604, USA; 2 Department of Molecular Genetics, Erasmus MC, Erasmus University Medical Center Rotterdam, 3000 CA Rotterdam, The Netherlands; 3 Oncode Institute, Erasmus MC, Erasmus University Medical Center Rotterdam, 3000 CA Rotterdam, The Netherlands

## Abstract

Protein–protein interactions regulate many essential enzymatic processes in the cell. Somatic mutations outside of an enzyme active site can therefore impact cellular function by disruption of critical protein–protein interactions. In our investigation of the cellular impact of the T304I cancer mutation of DNA Polymerase β (Polβ), we find that mutation of this surface threonine residue impacts critical Polβ protein–protein interactions. We show that proteasome-mediated degradation of Polβ is regulated by both ubiquitin-dependent and ubiquitin-independent processes via unique protein–protein interactions. The ubiquitin-independent proteasome pathway regulates the stability of Polβ in the cytosol via interaction between Polβ and NAD(P)H quinone dehydrogenase 1 (NQO1) in an NADH-dependent manner. Conversely, the interaction of Polβ with the scaffold protein X-ray repair cross complementing 1 (XRCC1) plays a role in the localization of Polβ to the nuclear compartment and regulates the stability of Polβ via a ubiquitin-dependent pathway. Further, we find that oxidative stress promotes the dissociation of the Polβ/NQO1 complex, enhancing the interaction of Polβ with XRCC1. Our results reveal that somatic mutations such as T304I in Polβ impact critical protein–protein interactions, altering the stability and sub-cellular localization of Polβ and providing mechanistic insight into how key protein–protein interactions regulate cellular responses to stress.

## INTRODUCTION

The vital importance of genome maintenance is underscored by the evolution of multiple DNA repair mechanisms, each of which functions on a specific type or class of damaged DNA. Of these, the base excision repair (BER) pathway plays a critical role in repairing base damage and DNA single-strand breaks that emerge from both endogenous and exogenous sources. Failure to repair such DNA lesions can lead to accumulation of DNA mutations and chromosome alterations. As such, defects in DNA repair pathways or proteins can predispose to cancer and disease onset (1). Such defects in DNA repair can arise from mutations in essential active site amino acid residues (2), as well as those critical for post-translational modifications (3), protein–protein interactions (4) or protein complex assembly or dis-assembly (5). This study focuses on somatic mutations found in the gene for DNA polymerase β (Polβ) and its impact on the BER pathway.

The BER pathway plays a major role in the repair of endogenous and exogenous DNA damage that induces alkylated bases, oxidatively modified bases, base deamination and DNA hydrolysis (6). Polβ is the primary DNA polymerase involved in BER and both its 5′deoxyribose phosphate (5′dRP) lyase and nucleotidyl transferase activities are important for BER (7,8). Mutations in Polβ are found in many human cancers and recently, as many as 75% of the tumors analyzed in a colon cancer cohort were found to bear mutations in the coding region or the UTR region of the *POLB* gene (9–11). Modification of key amino acid residues impacting the 5′dRP lyase and nucleotidyl transferase functions of Polβ impairs BER efficiency and results in increased sensitivity to many DNA damaging agents (7,8). In addition, mutations that alter the structure of Polβ can affect its activity (12,13), such as the R137Q variant that confers cell sensitivity to the alkylating agent methyl methanesulfonate (14) or the P242R mutant that predisposes the cell to genomic instability and transformation (15).

Polβ is critical for both the gap-tailoring and gap-filling functions of BER (7,8,16). Polβ is a bi-functional, two-domain, 39 kDa enzyme (17). The N-terminal 8-kDa domain of Polβ possesses 5′dRP lyase activity that removes the sugar-phosphate lesion (5′dRP) during BER. The 31-kDa polymerase domain of Polβ is responsible for gap-filling DNA synthesis during BER and resides within the C-terminus (17). As we and others have described, these repair functions of Polβ are promoted or enhanced via essential protein–protein interactions (18,19) as part of the suggested hand-off or baton mechanism of BER (20). Of these protein partners, Polβ interacts with X-ray repair cross complementing 1 (XRCC1) (21,22), flap endonuclease 1 (FEN1) (23,24), apurinic/apyrimidinic (AP) endonuclease 1 (APE1) (25), proliferating cell nuclear antigen (PCNA) (26) and p53 (27), among others. Many somatic mutations of Polβ have been identified (9), including those that may prevent critical protein–protein interactions, such as the R137Q mutation that disrupts the interaction of Polβ with PCNA (14).

Numerous studies have suggested that cellular homeostasis of Polβ protein levels is important for proper cellular function and genome maintenance. Low levels of Polβ increase cancer susceptibility (28,29), while overexpression of Polβ is associated with increased carcinogenesis (30–32). As such, protein degradation plays a central role in regulating many processes of DNA repair and the cellular response to DNA damage (33,34). As we have shown, part of the homeostatic regulation of the Polβ protein is mediated by its interaction with XRCC1, since ‘free’ Polβ (not bound to XRCC1) can be targeted for ubiquitylation and degradation (18). In other unrelated studies, it has been found that protein homeostasis can also be regulated by the core 20S proteasome, by a process that does not require ubiquitylation (35).

We have extended our studies on the homeostasis of Polβ to include cancer mutants that may trigger defects in key protein–protein interactions. In this report, we have focused on the T304I colon cancer mutation of Polβ (11). This mutation is located within the XRCC1 interaction domain, known as the V303 loop (21,36,37), and we show here that the Polβ(T304I) mutant is defective in its ability to form a heterodimer with XRCC1. Importantly, we find that the Polβ(T304I) protein is unstable, leading to enhanced degradation. We also show that proteasome-mediated degradation of Polβ is regulated by both ubiquitin-dependent and ubiquitin-independent processes via unique protein–protein interactions. The ubiquitin-independent proteasome pathway regulates the stability of Polβ in the cytosol, via an interaction between Polβ and NAD(P)H quinone dehydrogenase 1 (NQO1) in an NADH-dependent manner. Conversely, the interaction of Polβ with XRCC1 plays a role in the chromatin localization of Polβ and regulates the stability of Polβ via a ubiquitin-dependent pathway. Further, we find that oxidative stress promotes the dissociation of the Polβ/NQO1 complex, enhancing the interaction of Polβ with XRCC1. Our results reveal that somatic mutations such as T304I in Polβ mitigate protein–protein interactions, thereby regulating the stability and sub-cellular location of Polβ. Herein, we provide mechanistic insight into how key protein–protein interactions regulate cellular responses to stress.

## MATERIALS AND METHODS

### Materials

Heat-inactivated fetal bovine serum (FBS), geneticin, Precast 4–20% Tris-glycine gels, L-glutamine, antibiotic/antimycotic and penicillin/streptomycin were from Thermo Fisher Scientific (Waltham, MA). Talon metal affinity resin and puromycin were from Clontech Laboratories (Takara Bio USA, Inc.). Gentamycin, N-Ethylmaleimide (prepared as a 0.4 M stock solution in ethanol), Anti-Flag M2 affinity gel, cycloheximide (prepared as a 100 mM stock solution in DMSO), MG132 (prepared as a 100 mM stock solution in DMSO) and hydrogen peroxide solution (diluted in H_2_O) were from MilliporeSigma. McCoy’s 5A medium, Dulbecco’s modified Eagle’s medium (DMEM), α-MEM and minimal essential medium (MEM), as well as the Glutathione agarose, Pierce IP lysis buffer and RIPA buffer, were from Thermo Fisher Scientific. Dimethyl sulfoxide (DMSO) was from Fisher Biotech (Fair lawn, NJ). Fugene 6 transfection reagent and protease inhibitor cocktail tablets were from Roche (Indianapolis, IN). NADH was from Alfa Aesar Chemicals of Thermo Scientific (Tewksbury, MA). Mono-S 5/50 GL column, Superdex 200 increase 10 × 300 GL column and glutathione sepharose 4B were from GE Healthcare (Piscataway, NJ). All of the primers were synthesized and purified by Thermo Fisher Scientific (Waltham, MA).

### Lentiviral vectors for expression of EGFP, Polβ(WT) and Polβ mutants

Human Flag-tagged wild-type Polβ cDNA, Flag-Polβ(WT), was cloned into the pENTR/D-TOPO plasmid to create the pENTR/Flag-Polβ(WT) vector as described previously (38). Using this plasmid, K206A, K244A and T304I mutations were made with the QuickChange XL Site-Directed Mutagenesis kit. The primers used are listed in Supplementary Table S1. Once sequence verified, the open reading frames from each vector and of pENTR/EGFP were transferred into a Gateway-modified lentiviral vector (either pLVX-IRES-Puro, pLVX-IRES-Neo or pLVX-IRES-Hygro) by LR recombination, as we have described previously (39). All the vectors developed and used in this study are listed in Supplementary Table S2.

### Construction of the pGEX4T3 plasmids expressing GST fusion proteins in *Escherichia coli*

To express and purify recombinant proteins (NQO1, NQO1(Y128F), Polβ(WT) and Polβ mutants including K206A/K244A (DM), T304I, K206A/K244A/T304I, K72A, D256A, L301R/V303R/V306R (TM) and TM/DM), the *E. coli* expression plasmid pGEX-4T-3 was modified to encode each of the open reading frames listed and then sequence validated. To construct pGEX-4T-3 plasmids expressing the indicated proteins, primers with SalI and NotI restriction enzyme sites were designed and used for polymerase chain reaction (PCR) to amplify the cDNA of the corresponding proteins. The sequence of each PCR primer used is listed in Supplementary Table S1. Both the PCR product(s) and the pGEX-4T-3 plasmid were digested by SalI and NotI, and the fragments were purified by gel purification with the QiaQuick Gel Extraction kit (Qiagen). The fragments were ligated and transformed into BL21-CodonPlus-RP cells. The positive plasmids were then isolated and sent to Eurofins Genomics for sequence validation. The primers for sequencing are listed in Supplementary Table S1. The plasmids with the correct sequence were then used for protein expression and purification. The expression of the proteins in *E. coli* was then examined by Coomassie blue staining and immunoblot analysis (Supplementary Figure S1A).

### Cell culture and cell line development

HCT116 cells were cultured in McCoy’s media supplemented with 10% heat-inactivated FBS and Pen/Strep. LN428 cells were cultured in α-MEM supplemented with heat-inactivated FBS (10%), gentamycin (5 μg/ml), pen/strep/amphotericin and L-glutamine (2 mM). U2OS cells were cultured in DMEM supplemented with heat-inactivated FBS (5%) and Penn/Strep. T98G cells were cultured in MEM supplemented with heat-inactivated FBS (10%), gentamycin (5 μg/ml), penicillin (80 units/ml), streptomycin (80 μg/ml), amphotericin (32 μg/ml), sodium pyruvate (1 mM) and non-essential amino acids (0.1 mM). In most cases, cells transduced with an EGFP-expressing lentivirus were used as control. HCT116 cells, HCT116/Polβ-KO cells, LN428 cells and LN428/Polβ-KO cells expressing Flag-Polβ(WT) and Flag-Polβ mutants (including TM, K206A, K244A, K206A/K244A, TM/K206A/K244A, T304I, K244A/T304I and K206A/K244A/T304I) were developed by lentiviral transduction. The generation of lentiviral particles and the collection and isolation of lentiviral particles were performed as described previously (18,38). Stable cell lines were cultured in selection media for 1 week. Whole cell lysates (WCL) were prepared and analyzed by immunoblotting to determine the expression of the desired proteins. All of the cells were cultured at 5% CO_2_ and 37°C. All the cell lines developed and used in this study and their growth media are listed in Supplementary Table S3.

### Purification of recombinant proteins expressed in *E. coli*

For the binding assay (Open-SPR), the Polβ/NQO1 interaction *in vitro* assay and 20S proteasome *in vitro* degradation assay, we purified Polβ(WT), Polβ(T304I), NTD-XRCC1(1-151)-His, NQO1 and NQO1(Y128F) proteins. The protein purification scheme is shown in Supplementary Figure S1D. The procedure was performed as described previously (40,41) with some minor modifications as described: BL21-CodonPlus-RP cells expressing GST-Polβ(WT), GST-Polβ(T304I), GST-NQO1 and GST-NQO1(Y128F) were utilized for GST-tag protein expression. An overnight culture was added to fresh LB medium with 1:100 dilution. The cells were cultured until OD_600_ reached 0.6–1, then 0.4 mM isopropyl β-D-1-thiogalactopyranoside (IPTG) was added and cells were cultured overnight at 18°C. The cell pellet was collected by centrifugation at 5000 rpm for 10 min at 4°C. The cell pellet was then washed with lysis buffer (50 mM 4-(2-hydroxyethyl)-1-piperazineethanesulfonic acid (HEPES) pH 7.4, 500 mM NaCl, 1× Sigma protease inhibitor cocktail, 1 mM Dithiothreitol (DTT) and 1 mM EDTA). Cells were resuspended in lysis buffer and lysed by sonication (10 s on and 20 s off for 2 min). Cell lysates were then centrifuged at 10 000 rpm for 20 min at 4°C twice to collect the cell lysate supernatant. Cell lysate supernatant was mixed with 2 ml of glutathione sepharose 4B resin, washed with lysis buffer and the mixture was rotated overnight. The resin was washed with lysis buffer four times. Then, the resin was washed with 50 mM HEPES pH 7.4, 100 mM NaCl (buffer A) to exchange into a low salt buffer. To remove the GST-tag, the resin was resuspended with Buffer A and 0.5 ml of 1 mg/ml GST-TEV protease and incubated overnight at 4°C with rotation. The resin was poured into a column and the flow-through was collected. The column was washed again with Buffer A and the flow-through was collected.

For Polβ and its mutants, the flow-through was loaded onto a Mono-S column equilibrated with 10 column volumes of buffer A at a flow rate of 1 ml/min. The Mono-S column was washed with 10 ml Buffer A at a flow rate of 1 ml/min, then the Polβ protein was eluted with 0–100% Buffer B (50 mM HEPES pH 7.4, 1 M NaCl) 30 min and 100% Buffer B for another 20 min. The fractions containing Polβ were pooled and concentrated with Millipore Amicon Ultra-4 centrifuge filters. The concentrated fraction was loaded onto a gel filtration column (Superdex 200 increase 10/300 GL, GE Health) equilibrated in 50 mM HEPES pH 7.4, 150 mM NaCl. After the proteins were eluted, fractions were collected and concentrated. Purity was examined by sodium dodecylsulphate-polyacrylamide gel electrophoresis (SDS-PAGE) followed by Coomassie blue staining and immunoblot, as shown in Supplementary Figures S1F and S3F.

For NQO1 and NQO1(Y128F), the flow-though was concentrated with Millipore Amicon Ultra-4 centrifuge filters. The concentrated flow-through was loaded onto a gel filtration column. The procedure was performed as described for the purification of Polβ and its mutants above.

To purify NTD-XRCC1(1-151)-His, the plasmid pET21a-NTD-hXRCC1(1-151)-His was transformed into BL21 cells. The expression of the protein was induced by the addition of 1 mM IPTG. The His-tagged XRCC1 protein was purified by Talon Metal affinity resin (Clontech). The eluent containing the His-Tagged XRCC1 protein was further purified by gel filtration, as described above. The purification processes are summarized in Supplementary Figure S1D. Representative chromatographs are shown in Supplementary Figure S1E.

#### Cell extract preparation

For newly developed stable cell lines, the expression level of proteins was determined by immunoblot using WCL. WCL was prepared as described previously and quantified using a DC protein assay following the microplate protocol provided by the company with the DC protein assay kit (Bio-Rad) (18).

#### Immunoprecipitation (IP)

To study how the Polβ(T304I) mutation affects the interaction of XRCC1 with Polβ and how H_2_O_2_ treatment affects the Polβ/XRCC1 interaction, anti-Flag M2 affinity gel, Polβ (Clone61) antibody and XRCC1 antibodies were used to immunoprecipitate (IP) Flag-Polβ(WT) and Flag-Polβ mutants, endogenous Polβ and XRCC1, respectively. The IP was performed as described previously (18).

#### Immunoblot

Twenty to thirty micrograms of WCL or 5–10μl immunoprecipitated proteins were loaded on a precast 4–12% NuPAGE Tris-glycine gel, run for 2–3 h at 100–130 volts. The gel was transferred and the membrane was blotted with primary antibodies, as indicated. The information for the primary antibodies used herein is listed in Supplementary Table S4. After washing, Immun-Star Goat anti-mouse-HRP conjugate (Bio-Rad) or Immun-Star Goat anti-rabbit-HRP conjugate (Bio-Rad) secondary antibody was used. The membrane was illuminated and the bands were quantified using Image Lab (Bio-Rad).

### Surface plasmon resonance (OpenSPR) assay

Binding experiments were carried out in an OpenSPR localized surface plasmon resonance (LSPR) biosensor (Nicoya Lifesciences). The running buffer used throughout the experiment contains 25 mM HEPES pH 8.0, 150 mM NaCl, 200 mM MgCl_2_, 0.1% Tween 20 and 1 mM Tris(2-carboxyethyl)phosphine hydrochloride (TCEP). Polβ(WT) or Polβ(T304I) mutant was diluted in immobilization buffer (10 mM malate pH 6.0) at a final concentration of 50 μg/ml. The carboxyl sensor surface (Nicoya) was activated by injecting a mixture of 1-ethyl-3-(3-dimethylaminopropyl) carbodiimide (42) and N-hydroxysuccinimide (NHS) followed by immobilization of Polβ(WT) or Polβ(T304I) via covalent coupling. The free carboxyl groups were deactivated using blocking buffer (Nicoya). After achieving a stable baseline, the running buffer was injected for blank measurement followed by successive injections of buffer matched XRCC1 at concentrations of 10, 100 nM, 1, 2, 5 and 10 μM. The data were analyzed using TraceDrawer 1.8 and *K*_D_ values were calculated using the affinity model where the equilibrium dissociation constant, *K*_D_, is calculated from a response versus concentration plot using non-linear regression (*Y* = *B*_max_ * *c* / (*c* + *K*_D_)).

### Stability assay for Polβ(WT) and Polβ mutants

The stability assay of Polβ(WT) and the Polβ mutant proteins was carried out as described previously (18). The level of Polβ and mutants was determined by immunoblot, and the intensity of bands was quantified using Image Lab (Bio-Rad). To study the stability of Polβ(WT), Polβ(T304I) and Polβ(T304I/DM), cells were treated with 0.2 mM cycloheximide (Cyc) or Cyc plus 25 μM MG132 for 0, 2, 4 and 6 h. WCL was prepared and the level of Polβ(WT) or Polβ mutants was determined and quantified. The level of PCNA or α-tubulin was set as a loading control in these experiments.

### Quantitative RT-PCR analysis

Expression of mRNA for Polβ(WT) and Polβ mutants was measured by quantitative RT-PCR using an Applied Biosystems StepOnePlus system (39). Briefly, 80 000 cells were lysed and reverse transcribed using the Applied Biosystems Taqman® Gene Expression Cells-to-CT kit, as we have described previously (43). Analysis of mRNA expression was performed as per the instruction of the manufacturer (ΔΔ*C*_T_ method). Hs00160263_m1 (human Polβ) was used in the TaqMan Gene Expression Assay. Samples were run in triplicate and the results shown are the mean ± SD of all three analyses. The mRNA level of Polβ is then normalized to the expression of human β-actin (Hs99999903_m1).

### Determination of ubiquitylated Polβ in cells

HCT116 cells and LN428 cells expressing EGFP, Flag-Polβ(WT), Flag-Polβ(T304I), Flag-Polβ(K244A/T304I) or Flag-Polβ(T304I/DM) were used. Cells were transfected with 12 μg pCDNA-HA-ubiquitin with Promega Fugene HD. The Flag-Polβ and Flag-Polβ mutants were IP with M2 agarose, and the eluted IP products were probed with anti-HA antibody and Polβ(595) antibody to evaluate ubiquitylated Polβ (18).

### *In vitro* assay to determine the interaction of Polβ with NQO1—cell lysates

BL21-CodonPlus-RP cells expressing GST-Polβ and GST-NQO1 were utilized for GST-tag protein purification. The procedure describing the binding of the GST-tagged proteins to glutathione-agarose (Thermo Fisher Scientific) is described above (‘Purification of recombinant proteins expressed in *E. coli* ’). After the GST-tagged proteins (purified from 300 ml culture) were bound to the glutathione-agarose, the agarose/GST-tagged protein complex was washed with lysis buffer (see above). The agarose was then aliquoted into three fractions (1, 2 and 3), and each fraction was mixed with 1 ml of cell lysate prepared from a 1 × 150 mm dish of HCT116, LN428 or T98G cells, respectively. The mixture was incubated at 4°C overnight with rotation. The agarose was then washed 3–5 times with lysis buffer. After washing, each agarose preparation (1, 2 and 3) was separated into two fractions (1a, 1b, 2a, 2b, 3a, 3b). One set of fractions (1a, 2a, 3a) was then used to determine the total proteins captured by the glutathione-agarose/GST-protein complex. The second set of fractions (1b, 2b, 3b) was then incubated with GST-TEV protease overnight at 4°C to release Polβ and its mutants, allowing analysis of the proteins bound only to the glutathione-agarose/GST fragment. After the removal of the buffer from the glutathione-agarose, 100 μl of 2× Laemmli buffer (62.5 mM Tris–HCl, pH 6.8, 20% (w/v) glycerol, 2% SDS, 0.01% Bromophenol Blue) was added to (i) the glutathione-agarose/GST-protein complex or (ii) glutathione-agarose/GST fragment complex, followed by incubation in boiling water for 5 min to elute the bound proteins. The level of PARP1, XRCC1, TBP1 (26S proteasome), C2 (20S proteasome) and NQO1 in the elution was examined by immunoblot.

To study the effect of Polβ mutants on the interaction of Polβ with NQO1, 100 ml of BL21-CodonPlus-RP cells expressing GST-Polβ, GST-Polβ mutants, GST-NQO1, GST-NQO1(Y128F) or GST-EGFP were used and GST-proteins were isolated with glutathione-agarose. The glutathione-agarose was incubated with cell lysates from HCT116 cells for 4 h and the GST-tagged proteins were eluted with 2× Laemmli buffer by heating in boiling water. The level of bound PARP1, XRCC1, TBP1, C2, NQO1 and loaded Polβ and its mutants (GST) were examined by immunoblot. GST-EGFP served as a negative control.

To study the effect of dicumarol treatment on the interaction of Polβ with NQO1, 200 or 400 μM dicumarol in DMSO was used to treat HCT116, LN428, LN428/EGFP, LN428/Flag-Polβ, T98G and T98G/Flag-Polβ cells for 5 h. Then, WCL was prepared and quantified and the level of bound PARP1, XRCC1, Polβ, p53, NQO1 and PCNA was examined as above.

### Determination of the interaction of Polβ with NQO1—purified proteins

Glutathione-agarose was used to bind GST-Polβ(WT), GST-Polβ(T304I) and GST-Polβ(TM). The glutathione-agarose with the GST-tagged proteins was then incubated with 1 μg of purified NQO1 or NQO1(Y128F) in 1 ml of 50 mM HEPES pH 7.4 buffer with 150 mM NaCl, 20 μg/ml of bovine serum albumin (BSA) and 5 mM DTT overnight at 4°C. The agarose was washed with TBS five times. The bound proteins were eluted with 30 μl of 2× Laemmli sample buffer by boiling 5 min in water. The level of NQO1 and Polβ was examined by immunoblot.

### *In vitro* Polβ degradation assay mediated by the 20S proteasome

Purified Polβ(WT) and Polβ(T304I) recombinant proteins (200 ng each) were incubated with 500 ng of the 20S proteasome (Sigma) in the absence or presence of 0.5% DMSO, 50 μM MG132, 500 ng NQO1 or 500 ng NQO1 plus 5 mM NADH in 10 μl of reaction buffer (100 mM Tris–HCl pH 7.5, 150 mM NaCl, 5 mM MgCl_2_, 2 mM DTT, 20 μg/ml BSA, 10% glycerol) at 37°C for 1 h. After 1 h, 10 μl of 2× Laemmli sample buffer was added and the samples were boiled for 5 min in water. The level of the proteins Polβ, NQO1 and the 20S proteasome protein C2 was examined by immunoblot.

### XRCC1 and Polβ knockout by CRISPR/Cas9 in U2OS, T98G, HCT116 and LN428 cells

Guide RNAs (gRNAs) targeting XRCC1 exon 2 or exon 3 or targeting Polβ exon 1 were designed using the CRISPR Design Tool (44), and as described (45). The gRNAs for XRCC1 were cloned into pLentiCRISPRv2 (a gift from Feng Zhang) (46). The gRNAs for Polβ were cloned into pLentiGuide and pLentiCRISPR-GFP. The sequence of each gRNA target sequence is listed in Supplementary Table S1 and the plasmids are listed in Supplementary Table S2. The experiment was performed as described (46,47). Briefly, the U2OS, T98G, HCT116 and LN428 cell lines were transduced by lentivirus prepared from the corresponding vectors (see Supplementary Table S2) (18,48). Cells were selected by culturing in puromycin-supplemented media (1 μg/ml) for 5 days after transduction for 48 h. Cells were then seeded for selection of single cell clones and knockout was confirmed by immunoblot. The gene knockout was also confirmed by DNA sequencing of the targeted exon 2 or exon 3 (XRCC1) using primer pairs XRCC1-KO-2-fw/XRCC1-KO-2-re or XRCC1-KO-3-fw/XRCC1-KO-3-re, or by DNA sequencing of the targeted exon 1 (Polβ) with primers Polβ-KO-1-Fw and Polβ-KO-1-Re, respectively. The sequence of the PCR primers used to perform PCR and DNA sequence analysis is listed in Supplementary Table S1.

### NAD^+^/NADH measurements

The level of NAD^+^ and NADH in LN428 cells treated with 0, 150, 200 and 250 μM H_2_O_2_ for 3 h was measured by the Enzychrome NAD^+^/NADH assay kit (BioAssay Systems) as we have described previously (48). Briefly, LN428 cells were seeded in 6-well plates at a density of 2 × 10^5^ cells per well. Twenty-four hours later, cells were treated with the indicated concentration of H_2_O_2_ for 3 h. Following treatments, cells were harvested and a suspension of 2 × 10^5^ cells was divided in half for measuring NAD^+^ and NADH, respectively. Cell pellets were homogenized using plastic pestles and the extraction of NAD^+^ and NADH was performed as per the manufacturer’s protocol using the lysis buffers provided. Extracts were heated at 60°C for 5 min and neutralized with the extraction buffer. Samples were spun down and the supernatant was immediately used for measurements of NAD^+^/NADH content using a Molecular Devices VersaMax™ tune-able plate reader at 565 nm wavelength.

### Isolation of cytosolic, nucleoplasmic and chromatin fractions

To study the distribution of Polβ in cells (LN428 expressing Flag-Polβ(WT), Polβ(T304I) or Polβ(TM), as well as U2OS, T98G and LN428 cells with or without XRCC1), fractions of cytosolic, nucleoplasmic (soluble nuclear proteins) and chromatin-bound proteins were isolated. Cytosolic, nucleoplasmic and chromatin fraction isolation was performed as follows: cells were cultured until the plates reached 80–90% confluence (100 mm plates). Media was removed and the cells were then washed twice with cold phosphate-buffered saline, and 300 μl of cytoplasmic lysis buffer (10 mM Tris–HCl pH 8.0, 0.34 M Sucrose, 3 mM CaCl_2_, 2 mM Mg acetate, 0.1 mM EDTA, 0.5% Nonidet P-40, protease inhibitor) was added to plates. Cells were then scraped and the cell/buffer mixture was transferred to 2 ml Eppendorf tubes. Cells were incubated for 15 min on ice and then were centrifuged for 10 min at 4000 rpm (4°C). The supernatant is the cytoplasmic fraction and was collected and prepared for immunoblot.

The pellet (nuclei) was carefully washed with 300 μl of wash buffer (10 mM Tris–HCl pH 8.0, 0.34 M sucrose, 3 mM CaCl_2_, 2 mM Mg acetate, 0.1 mM EDTA, protease inhibitor) and then the buffer was removed with a pipette without resuspension or centrifugation. The nuclei were lysed in 100 μl nuclear lysis buffer (20 mM HEPES pH 8.0, 3 mM EDTA, 10% glycerol, 150 mM potassium acetate, 1.5 mM MgCl_2_, 0.1% Nonidet P-40, protease inhibitor) and incubated on ice for 30 min. The nuclear lysate was centrifuged at 13 000 rpm for 10 min (4°C). Here, the supernatant is the nucleoplasmic fraction and was collected and prepared for immunoblot.

The pellet (chromatin fraction) was washed with 300 μl nuclear lysis buffer and then with 750 μl nuclease incubation buffer (150 mM HEPES pH 8.0, 10% glycerol, 50 mM potassium acetate, 100 mM KCl, 1.5 mM MgCl_2_, protease inhibitor) carefully with a pipette without resuspension or centrifugation. The pellet was then re-suspended in 75 μl of nuclease incubation buffer with 1.5 μl benzonase and incubated for 15 min at 37°C and mixed to re-suspend every 5 min. The resuspension was centrifuged at 13 000 rpm for 15 min (4°C). Here, the supernatant is the chromatin fraction and was collected and prepared for immunoblot.

The level of Polβ, XRCC1, PARP1, NQO1 and α-tubulin in each of the fractions was determined by immunoblot. The relative level of Polβ in the cytosolic, nucleoplasmic and chromatin fractions was quantified using Image Lab (Bio-Rad). For LN428 cells expressing Flag-Polβ(WT) and Polβ(T304I) or for U2OS, T98G and LN428 cells with or without XRCC1, the relative level of Polβ in each isolated fraction was normalized to the corresponding cytosolic fraction.

To evaluate how H_2_O_2_ treatment affects the location of Polβ, LN428 or T98G cells were treated with H_2_O_2_ (150 μM) for 0, 2, 4, 6, 12 or 24 h and the cytosolic and chromatin fractions were isolated. The relative level of Polβ, XRCC1 and PARP1 was quantified using Image Lab (Bio-Rad) and the ratio of Polβ/PARP1 and XRCC1/PARP1 was calculated. To evaluate whether H_2_O_2_ treatment stimulates the expression of Polβ in LN428 or T98G cells, the cells were treated with H_2_O_2_ (150 μM) for 0, 2, 4, 6, 12 or 24 h and then WCL were prepared. The relative level of Polβ, XRCC1, PARP1 and α-tubulin was examined by immunoblot. The level of Polβ and α-tubulin was quantified using Image Lab (Bio-Rad) and the ratio of Polβ/α-tubulin was calculated.

### *In vitro* Polβ degradation assay following dicumarol treatment

Dicumarol was dissolved in DMSO. LN428, HCT116, T98G, LN428/Flag-Polβ(WT) and T98G/Flag-Polβ(WT) cells were treated with dicumarol (200 or 400 μM) for 5 h. Then, WCLs were prepared and the level of Polβ, p53 (positive control), PARP1, XRCC1, NQO1 and PCNA was examined by immunoblot. The level of Polβ was quantified using Image Lab (Bio-Rad) and the relative level of Polβ was calculated as the ratio of Polβ/PCNA.

### Immunofluorescence (IF) assay

To determine whether H_2_O_2_ treatment promotes the nuclear translocation of Polβ, 2 × 10^5^ LN428 cells were seeded into 35 mm dishes with #1.5 cover glass bottoms (World Precision Instruments, FD35-100) for 24 h. Cells were then treated with H_2_O_2_ (150 μM) for 24 h. Control and H_2_O_2_ treated cells were then fixed with 95% cold methanol for 15 min, permeabilized with 0.1% Triton X-100 for 15 min and blocked with 2% bovine serum albumin for 45 min. Cells were then incubated with mouse anti-Polβ antibody (Clone 61) at 1:100 dilution for 2 h. After washing, cells were incubated with Alexa Fluor 488 conjugated goat anti-mouse IgG (Life Technologies, Inc.) at 1:1000 dilution for 1 h. After washing, cells were mounted using Prolong Gold anti-fade reagent with DAPI (Life Technologies, Inc.) and a #1.5 coverslip. Cells were imaged with a Nikon A1r confocal microscope using a Plan Apo λ 60× objective (NA = 1.4). For each field, an image stack through the *Z*-plane was collected to fully sample cells. Images were quantified using a custom macro written for NIS-Elements (Laboratory Imaging). Briefly, a maximum intensity projection of each image stack was generated, and the DAPI stain was used to define each nucleus as a region of interest (ROI). ROIs touching the image border were removed, and then mean Polβ intensity per nucleus was measured; 100–200 cells per condition were analyzed. Statistical analysis was performed using Student’s *t*-test.

To determine the relative distribution of Polβ in the nucleus and cytoplasm, U2OS/Cas9, U2OS/XRCC1-KO.2.1, U2OS/XRCC1-KO.3.1, U2OS/Polβ-KO.2.1, T98G/Cas9, T98G/Polβ-KO.2.2, T98G/XRCC1-KO.2.4 and T98G/XRCC1-KO.3.4 cells were seeded into cover glass bottom dishes as above and cultured for 24 h. Cells were fixed with 4% paraformaldehyde for 15 min, permeabilized with 0.1% Triton X-100 for 15 min and blocked with 2% bovine serum albumin for 45 min. Cells were then incubated with mouse anti-Polβ antibody (Clone 61) at 1:100 dilution and rabbit anti-XRCC1 antibody at 1:500 dilution for 2 h. After washing, cells were incubated with Alexa Fluor 488 conjugated goat anti-mouse IgG and Alexa Fluor 568 conjugated goat anti-rabbit IgG at 1:1000 dilution, together with Alexa Fluor 647 conjugated phalloidin at 1:40 dilution for 1 h (Life Technologies, Inc.). After washing, cells were mounted using Prolong Gold anti-fade reagent with DAPI (Life Technologies, Inc.) and a #1.5 coverslip. Cells were imaged with a Nikon A1r confocal microscope using a Plan Apo λ 60× objective (NA = 1.4). For each field, an image stack through the *Z*-plane was collected to fully sample cells. Cells were quantified using a custom macro written for NIS-Elements (Laboratory Imaging). Briefly, a maximum intensity projection of each image stack was generated, and the DAPI and phalloidin stains were used to define the nuclear and cytoplasmic components, respectively. For each image field, mean Polβ intensity for each compartment was measured and exported, and used to determine nuclear/cytoplasmic ratios and whole cell mean intensities (nuclear + cytoplasmic mean values). Nine image fields were analyzed, yielding 100–200 cells per condition. Statistical analysis was performed using one-way ANOVA followed by Tukey’s multiple comparison test.

### Statistical analysis

All data are shown as the mean ± standard deviation from 3 to 4 independent experiments. Student’s *t*-test was used for comparisons between two groups. For multiple comparisons, one-way or two-way ANOVA was used, followed by either Tukey’s or Dunnett’s multiple comparison test. Statistical analysis was performed using GraphPad PRISM.

## RESULTS

### DNA polymerase β colon cancer mutant T304I disrupts Polβ/XRCC1 complex formation and promotes Polβ ubiquitylation and degradation

**Figure 1.**
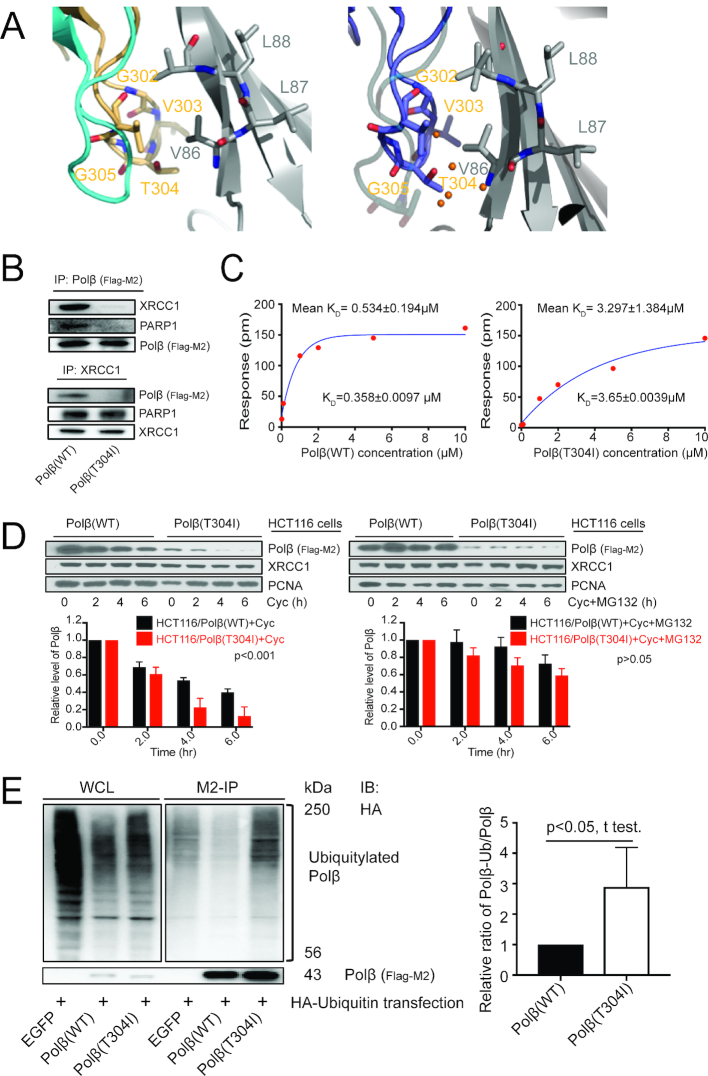
The T304I cancer mutation of Polβ disrupts the Polβ/XRCC1 interaction and induces the ubiquitylation and degradation of Polβ. (**A**) Polβ(T304I) location and the structure of Polβ interacting with XRCC1 shown in the reduced (left) and oxidized (right) form. Left panel: Cyan: 2FMS; Gray: XRCC1 in 3K75 (Polβ bound to reduced XRCC1); Wheat: Polβ in 3K75 (Polβ bound to reduced XRCC1). Right panel: Cyan: 2FMS; Gray: XRCC1 in 3lqc(Polβ: oxidized XRCC1); Slate: Polβ in 3K75(Polβ: oxidized XRCC1); Orange spheres: Water molecules. (**B**) Polβ(T304I) mutation disrupts the Polβ/XRCC1 interaction in HCT116 cells. Top panel: Flag (M2) immunoprecipitation (IP) followed by an XRCC1 immunoblot (IB) shows that Polβ(T304I) immunoprecipitates less XRCC1 than does Polβ(WT). Bottom panel: XRCC1-IP followed by a Flag (M2) IB shows that XRCC1 immunoprecipitates less Polβ(T304I) than Polβ(WT). The level of PARP1 binding to XRCC1/Polβ complex was also examined. The blots shown are an analysis of lysates from HCT116 cells (10 μl of IP eluates was loaded per lane). (**C**) Analysis of the interaction between Polβ and XRCC1 by OpenSPR shows that the Polβ(T304I) mutation (right) decreases the binding affinity (2.38–12.48 fold; mean = 6.18) to XRCC1 as compared to Polβ(WT) (left). The complete set of raw data (sensograms) and the repeat experimental datasets are shown in Supplementary Figure S1G and H. (**D**) Polβ(T304I) mutation induces the degradation of Polβ. Top panel: a representative immunoblot image of cycloheximide (Cyc) treatment resulting in the enhanced degradation of Polβ(T304I) in HCT116 cells (left panel). Treatment with the proteasome inhibitor MG132 stabilizes the level of the Polβ(T304I) protein (right panel). The immunoblot from two independent experiments are shown in Supplementary Figure S1I. Bottom panels: The relative level of Polβ and of PCNA was determined by densitometry and was quantified using Image Lab (Bio-Rad) and the ratio of band densitometry of Polβ/PCNA is shown. The ratios for each cell line at time 0 were normalized to 1 (25 μg of WCL was loaded per lane). Plots show the mean ± SD of three independent experiments. The relative level of Polβ(WT) was compared to Polβ(T304I) in HCT116 cells treated with Cyc (*P* < 0.001) or Cyc+MG132; (*P* > 0.05), as determined by regular two-way ANOVA. (**E**) Polβ(T304I) mutation promotes enhanced ubiquitylation of Polβ in HCT116 cells. HCT116 cells expressing EGFP, Flag-Polβ(WT) or Flag-Polβ(T304I) were transiently transfected with pcDNA-HA-ubiquitin and the Flag-tagged proteins were immunoprecipitates with Flag-M2 agarose. The ubiquitylated form of Polβ was examined by immunoblot using an HA antibody, as shown. The relative level of ubiquitylated Polβ (WT or T304I) was determined by densitometry and was quantified using ImageJ and the relative level of ubiquitylated Polβ was calculated by determining the ratio of ubiquitylated Polβ/loaded Polβ, as shown in the plot to the right (10 μl of IP eluates was loaded into each lane). The immunoblot from three additional independent experiments are shown in Supplementary Figure S1J; *P* < 0.05, unpaired *t*-test was used for the statistical analysis.

DNA polymerase β (Polβ) and XRCC1 form a tight heterodimeric complex via an interaction between the C-terminal domain of Polβ and the N-terminal domain of XRCC1 (21,36,37). Amino acid residues P300 to E309 on Polβ form the V303 loop, a domain that interfaces with XRCC1 via a hydrophobic pocket spanning amino acid residues F67 and V86 on XRCC1 (21,37). In our previous report, we demonstrated that disrupting the Polβ/XRCC1 heterodimer by mutating residues on Polβ within this V303 loop impacts the stability of Polβ, inducing its ubiquitylation and proteasome-mediated degradation (18). Several studies have demonstrated a high percentage of Polβ mutations in cancer (49–51). More recently, Sweasy *et al.* found as many as 75% of the tumors analyzed in a colon cancer cohort bear mutations in the coding region or the UTR regions of the *POLB* gene (11). One of these colon cancer mutations is located within the V303 loop, the T304I mutation, and is found in colon cancer tissue but not in the corresponding normal tissue (11) (Figure 1A). The structure of the Polβ/XRCC1 heterodimer (3LQC; the oxidized form of XRCC1) shows that the water molecules (orange spheres) form a network of H-bond interactions in the vicinity of residue T304, whereas the reduced XRCC1 structure (3K75, left panel) does not have water molecules built into the model owing to lower resolution of the structure. From the location of the water molecules in the structure, it can be surmised that mutation of T to I for residue 304 in Polβ may displace some of these H-bond interactions, suggesting that the Polβ(T304I) mutation may interfere with or even disrupt water-mediated interactions at the interface with XRCC1 (Figure 1A).

To determine whether the T304I mutation disrupts the Polβ/XRCC1 interaction *in vivo* and *in vitro*, we used lentiviral transduction to modify the colon cancer cell line HCT116, creating stable cell lines that express either Flag-Polβ(WT) or Flag-Polβ(T304I). The expression of Flag-Polβ(WT) and Flag-Polβ(T304I) had no effect on the level of other BER-related proteins such as PARP1, XRCC1, PCNA and HSP90 (Supplementary Figure S1B). Our earlier studies revealed that Polβ mutants unable to bind XRCC1 are unstable and present with a lower basal protein level in human cells (18). Consistent with these findings, the basal level of the Flag-Polβ(T304I) protein is reduced as compared to the Flag-Polβ(WT) protein while the mRNA level of Flag-Polβ(T304I) and Flag-Polβ(WT) are comparable (Supplementary Figure S1B and C). Analysis of HCT116 cell lysates by immunoprecipitation/immunoblot (IP/IB) using either the Flag-Ab (M2) or the XRCC1-Ab confirms the strong heterodimer formation of Flag-Polβ(WT) protein with endogenous XRCC1, whereas the T304I mutation of Polβ disrupts the formation of the Polβ/XRCC1 complex (Figure 1B). To further confirm that the Polβ(T304I) mutation disrupts the Polβ/XRCC1 interaction, we used an OpenSPR (surface plasmon resonance) assay to calculate the binding affinities of the Polβ(WT)/XRCC1 and Polβ(T304I)/XRCC1 interactions. The N-terminal fragment of XRCC1 [NTD-XRCC1(1-151)-His] and the WT and mutant forms of Polβ [Polβ(WT) and Polβ(T304I)] were expressed in *E. coli* and purified (Supplementary Figure S1A and D–F). The OpenSPR assay revealed that the binding affinity of Polβ(WT) to XRCC1 ranges from 2.38- to 12.48-fold higher than Polβ(T304I), as indicated by the *K*_D_ values, with a mean *K*_D_ differential of 6.18 (Figure 1C and Supplementary Figure S1G–H). This confirms that the Polβ(T304I) mutation perturbs the Polβ/XRCC1 interaction, consistent with an earlier report showing purified His-tagged Polβ(T304I) loses its interaction with XRCC1 *in vitro* (52).

The stability of Polβ depends on complex formation with XRCC1 (18). We hypothesized that the low basal level of Flag-Polβ(T304I) in HCT116 cells is the result of ubiquitylation and degradation via the proteasome pathway. To avoid the potential interference of endogenous Polβ in HCT116 cells, we developed HCT116/Cas9 control and HCT116/Polβ-KO cells (Supplementary Figure S1B, middle panel). With these, we then expressed EGFP, Flag-Polβ(WT) or Flag-Polβ(T304I) in the HCT116/Polβ-KO cells by lentiviral transduction (Supplementary Figure S1B, bottom panel and Supplementary Tables S2, S3 and S5). As shown, the Flag-Polβ(WT) protein is more stable than Flag-Polβ(T304I) in the presence of the protein synthesis inhibitor cycloheximide (Cyc), while treatment with the proteasome inhibitor MG132 stabilizes both Polβ(WT) and Polβ(T304I) proteins (Figure 1D and Supplementary Figure S1I and J). The presence of endogenous Polβ has no effect on the stability of the expressed Flag-Polβ(WT) and Flag-Polβ(T304I) proteins (Figure 1D and Supplementary Figure S1I and J). Further, we find enhanced ubiquitylation of Flag-Polβ(T304I) when expressed in HCT116 cells, as compared to the Flag-Polβ(WT) protein (Figure 1E and Supplementary Figure S1K).

### Polβ(T304I) stability is mediated by a ubiquitin-independent proteasome pathway

**Figure 2.**
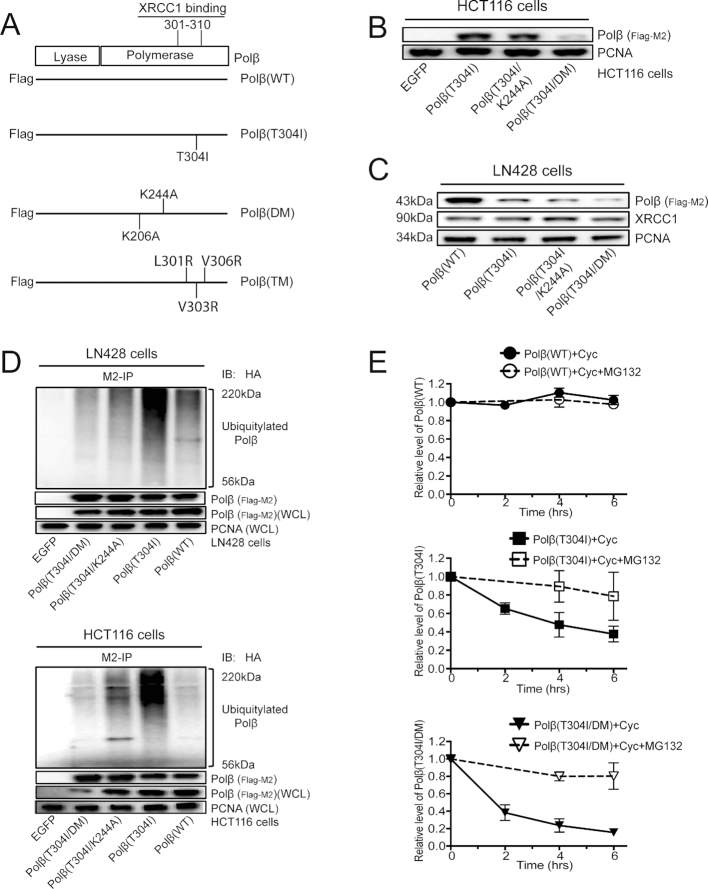
Blocking ubiquitylation does not promote the stability of the cancer mutant protein Polβ(T304I). (**A**) A scheme depicting the domains of Polβ and the mutations in Polβ used in this study. The vectors used express Polβ with an N-terminal Flag-tag (DYKDDDDK). The wild-type protein is designated WT; the focus of this study is the T304I mutant, originally identified in colon cancer (11); the double mutant whereby the lysine residues targeted for ubiquitylation have been changed to alanine (K206A/K244A) is denoted as DM (18); the triple mutant or separation of function mutant of Polβ that does not bind XRCC1 (L301R/V303R/V306R) is denoted as TM (18). Compound mutants were also developed, as listed in Supplementary Table S2. (**B**) Immunoblot showing the basal level of Polβ(T304I) expressed in HCT116 cells and with alanine mutations in the ubiquitylation sites K244 and K206. The double mutation (K206A/K244A) is denoted as DM. The level of PCNA is shown as a loading control. (**C**) Immunoblot showing the basal level of Polβ(WT) and Polβ(T304I) expressed in LN428 cells and Polβ(T304I) with alanine mutations in the ubiquitylation sites K244 and K206. The double mutation (K206A/K244A) is denoted as DM. The level of XRCC1 is also shown, with PCNA as a loading control. (**D**) Immunoblot showing enhanced ubiquitylation of Polβ(T304I), as compared to Polβ(WT), in LN428 cells (top) and HCT116 cells (bottom). Also shown is the reduced ubiquitylation of Polβ(T304I) with alanine mutations in the ubiquitylation sites K244 and K206. The double mutation (K206A/K244A) is denoted as DM. Total Polβ levels in the WCL are evaluated by immunoblot using the anti-Flag (M2) antibody. The level of PCNA in the WCL is shown as a loading control. (**E**) Plots denoting the basal level of Polβ(WT), Polβ(T304I) and Polβ(T304I/DM) expressed in LN428 cells in the presence of cycloheximide (Cyc) or Cyc + MG132 for the times indicated (the double mutation K206A/K244A is denoted as DM). The images of three independent experiments are shown in Supplementary Figure S2C. The relative level of Polβ treated with Cyc or Cyc + MG132 was determined by densitometry and was quantified using Image Lab (Bio-Rad) and calculated as the ratio of Polβ/PCNA. Results indicate the mean ± SD of three independent experiments.

Polβ is ubiquitylated on two lysine residues, K206 and K244, leading to proteasomal-mediated degradation (18). When expressed in LN428 cells, modification of both lysine residues to alanine (K206A/K244A, denoted as DM) stabilizes both the WT isoform of Polβ, Flag-Polβ(WT), as well as the separation-of-function mutant of Polβ that does not bind XRCC1 (L301R/V303R/V306R, denoted as TM), Flag-Polβ(TM), as we have reported (18). Similarly, the DM mutations increase the basal level of Flag-Polβ(WT) and Flag-Polβ(TM) protein when expressed in HCT116 cells (Supplementary Figure S2A). However, the K244A and DM mutations were found to decrease the basal level of the Flag-Polβ(T304I) protein in HCT116 and LN428 cells (Figure 2A–C), whereas qRT-PCR analysis shows that there is no significant difference in mRNA expression (Supplementary Figure S2B). This may suggest that the T304I mutation alters the target site for ubiquitylation. To that end, we evaluated whether Flag-Polβ(T304I) shows an elevated level of ubiquitylation and if the K244A or DM mutation blocks ubiquitylation of Polβ(T304I). As expected, since it has reduced binding to XRCC1, the Flag-Polβ(T304I) protein is highly ubiquitylated when expressed in LN428 or HCT116 cells (Figure 2D). Further, K to A modification of the ubiquitin target lysine residues 206 and 244 blocks the ubiquitylation of Flag-Polβ(T304I) in both LN428 cells and HCT116 cells (Figure 2D). However, the basal level of Flag-Polβ(T304I) is very low, prompting an analysis of its stability. A cycloheximide chase assay confirms that the DM mutation of the T304I mutant, Flag-Polβ(T304I/DM), does not block the enhanced turnover of the protein, while MG132 treatment partially or completely prevents the degradation of Flag-Polβ(T304I) and Flag-Polβ(T304I/DM) when expressed in LN428 or HCT116 cells (Figure 2E and Supplementary Figure S2C and D). Together, these findings suggest that the enhanced turnover of Flag-Polβ(T304I) in LN428 and HCT116 cells may be mediated by a ubiquitin-independent proteasomal degradation pathway.

### An interaction with NQO1 regulates the NADH-dependent and ubiquitin-independent proteasome-mediated degradation of Polβ

**Figure 3.**
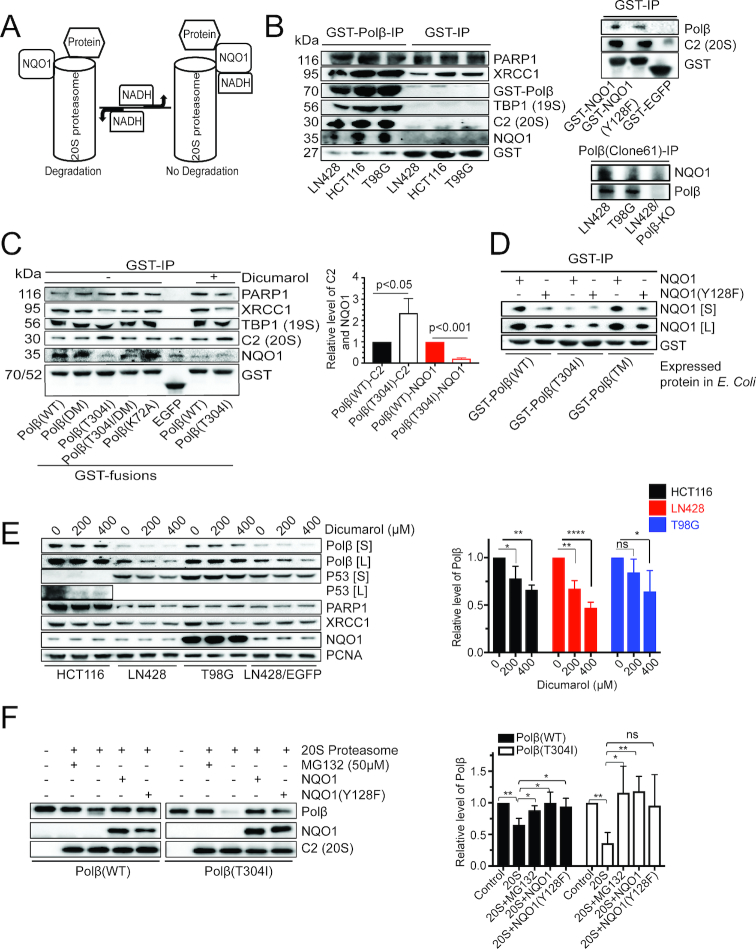
NADH regulates the binding and interaction of Polβ and NQO1. (**A**) Illustration depicting the interaction of target proteins with NQO1 to mediate ubiquitin-independent proteasome degradation. (**B**) Polβ interacts with NQO1. (Left panel) The representative immunoblot shows the proteins in LN428, HCT116 and T98G cell lysates binding to GST-Polβ, including NQO1, XRCC1, PARP1 and the 20S and 19S proteins C2 and TBP1, respectively, as compared to GST alone. (Top right panel) The immunoblot shows that GST-NQO1 and GST-NQO1(Y128F), but not GST-EGFP, bind to Polβ in cell lysates from LN428 cells. (Bottom right panel) The immunoblot shows that endogenous Polβ binds to endogenous NQO1 in LN428 and T98G cells. For all lanes, 10 μl of IP eluates were loaded, separated by SDS-PAGE and probed by immunoblot. However, to probe for the level of loading, 0.5 μl of the IP eluate was loaded into each lane and evaluated by IB with the anti-GST Ab. (**C**) The cancer mutant Polβ(T304I) has reduced binding to NQO1 and dicumarol treatment disrupts the interaction between Polβ and NQO1. Glutathione-agarose was mixed with cell lysates prepared from BL21-Codonplus-RP cells expressing GST-Polβ(WT) and its mutants and GST-EGFP to bind the indicated GST-tagged proteins. Glutathione-agarose/GST-Polβ(WT) (or Glutathione-agarose bound to GST-Polβ mutants or GST-EGFP, as indicated) was incubated with cell lysates prepared from LN428 cells or cells treated with 400 μM dicumarol for 5 h. Shown is an immunoblot indicating the level of bound NQO1, PARP1, XRCC1, TBP1 or C2. The images of two additional independent experiments are shown in Supplementary Figure S3A. The level of C2, NQO1 and Polβ (anti-GST) was determined by densitometry and quantified using Image Lab (Bio-Rad). The ratio of C2 to Polβ (*P* < 0.05) and the ratio of NQO1 to Polβ (*P* < 0.001) was calculated and plotted (right panel); Student’s *t*-test. (**D**) Polβ interacts with NQO1 *in vitro*. Glutathione-agarose/GST-Polβ(WT) (or Glutathione-agarose bound to GST-Polβ mutants, as indicated) was incubated with recombinant, purified NQO1 or NQO1(128F). Shown is an immunoblot indicating the level of bound NQO1; [*S*] = short exposure time; [*L*] = long exposure time. (**E**) Dicumarol treatment induces the degradation of endogenous Polβ and p53 (positive control) in human cells. The level of Polβ, p53, PARP1, XRCC1, NQO1 and PCNA in WCL was determined by immunoblot analysis of cell lysates prepared from control cells or after treatment with dicumarol; 200 or 400 μM, 5 h (the cell lines as indicated). The images of two additional independent experiments are shown in Supplementary Figure S3C. [*S*] = short exposure time; [*L*] = long exposure time. Quantitation summary of all three blots shown on the right. * *P* < 0.05, ** *P* < 0.01, **** *P* < 0.0001, ns: *P* > 0.05; compared to untreated cells. One-way ANOVA with Dunnett’s multiple comparisons test was used for the plot shown. (**F**) NQO1 protects Polβ from 20S proteasome *in vitro*. Purified Polβ(WT) or Polβ(T304I) (200 ng) was incubated with 20S proteasome alone or in the presence of DMSO (0.5%), MG132 (50 μM), NQO1 (500 ng) or NQO1 (500 ng) plus 5 mM NADH, at 37°C for 1 h. Shown is an immunoblot indicating the level of Polβ, NQO1 or 20S proteasome (C2). The images of two additional independent experiments are shown in Supplementary Figure S3G. The level of Polβ was determined by densitometry and was quantified using Image Lab (Bio-Rad). The ratio of Polβ(WT) or Polβ(T304I) ± the components listed was calculated and plotted (right panel); * *P* < 0.05, ** *P* < 0.01 and ns: *P* > 0.05. One-way ANOVA with Dunnett’s multiple comparisons test was used for the plot shown.

The ubiquitin-independent proteasomal degradation pathway is mediated by the 20S proteasome. There are several proposed regulatory mechanisms of this pathway, including disassembly of the proteasome, gene regulation and interaction with regulatory proteins (35). Proteins known to regulate the function of the 20S proteasome include the molecular chaperone heat shock protein 90 (HSP90), the DNA damage signaling protein poly-ADP-ribose polymerase 1 (PARP1) and NAD(P)H quinone dehydrogenase 1 (NQO1) (35). We have demonstrated that HSP90 does not interact with Polβ (18) and although PARP1 binds to XRCC1 (19), we do not find a direct interaction between Polβ and PARP1. Further, we show herein that the T304I mutation of Polβ disrupts the interaction with XRCC1, minimizing the level of PARP1 among the proteins bound to Polβ(T304I) (Figure 1B and Supplementary Figure S3A). As NQO1 has been shown to regulate the degradation of p53, ODC, Hif-1 and other proteins mediated by the 20S proteasome (35,53–55) (Figure 3A), we hypothesized that NQO1 may similarly regulate the degradation of Polβ(T304I).

We find that GST-Polβ interacts with endogenous NQO1 (Figure 3B, left panel) when probing cell lysates from HCT116, LN428 and T98G cells. Conversely, GST-NQO1 binds to endogenous Polβ and endogenous Polβ binds to endogenous NQO1, confirming the interaction between the two proteins (Figure 3B, right panel). This prompted us to investigate how the Polβ(T304I) mutation affects this interaction. Using GST-Polβ and a series of GST-Polβ mutants (Supplementary Table S2), including DM, T304I, T304I/DM and K72A, we evaluated how these mutations in Polβ affect the interaction with NQO1 and the related 20S proteasome (C2) and 19S proteasome (TBP1) proteins. Here, we find that GST-Polβ(T304I) binds less NQO1, as compared to GST-Polβ(WT), and has a greater affinity for the 20S proteasome (C2), when probing lysates of LN428 or T98G cells (Figure 3C and Supplementary Figure S3A and B). We next purified recombinant NQO1 and NQO1(Y128F) (Supplementary Figure S3F) and show, using an *in vitro* protein binding assay, that GST-Polβ(T304I) binds less NQO1 than does GST-Polβ(WT) or GST-Polβ(TM) (Figure 3D). Interestingly, the TM mutant (L301R/V303R/V306R), Polβ(TM), also disrupts the Polβ/XRCC1 interaction (18) yet we find that this mutation in Polβ does not affect the Polβ/NQO1 interaction. This suggests that the interaction of Polβ with NQO1 is not regulated by the status of the Polβ complex with XRCC1 (bound to or free of XRCC1) but instead is impacted by the presence of the cancer mutation, T304I.

As per current models, the regulation of the 20S proteasome function by NQO1 is NADH-dependent (53,54,56) (Figure 3A). We therefore performed pharmacologic and genetic experiments to address whether NADH mediates the Polβ/NQO1 interaction. Dicumarol (400 μM), a compound that specifically blocks NADH binding to NQO1 (57), disrupts the interaction of Polβ with NQO1 (Figure 3C) and promotes the degradation of endogenous Polβ and p53 (positive control) in a dose-dependent manner in HCT116, LN428 and T98G cells (Figure 3E and Supplementary Figure S3D). Further, we found that dicumarol treatment promotes the degradation of Flag-Polβ(WT) when overexpressed in LN428 and T98G cells (Supplementary Figure S3E). To confirm that the interaction of Polβ with NQO1 stabilizes Polβ, we depleted NQO1 in LN428 cells by shRNA (KD). We found that depletion of NQO1 in LN428 cells (LN428/NQO1-KD) results in lower basal levels of Polβ (Supplementary Figure S3C). Next, we tested the interaction of Polβ with NQO1(WT) as well as the mutant protein NQO1(Y128F), containing a mutation in the NADH-binding site (58). Less Polβ is bound to NQO1(Y128F) than to NQO1(WT) in an *in vitro* GST-pulldown assay (Figure 3D). Together, these data suggest that NQO1 mediates ubiquitin-independent degradation of Polβ in an NADH-dependent manner. To further test if the ubiquitin-independent proteasome degradation of Polβ was regulated by the Polβ/NQO1 interaction, we next performed an *in vitro* degradation assay. Here, we find that Polβ(T304I) is degraded more effectively than Polβ(WT) by the 20S proteasome when evaluated *in vitro*. In addition, the proteasome inhibitor MG132 or the addition of NQO1 impedes the degradation of both Polβ(T304I) and Polβ(WT) (Figure 3F and Supplementary Figure S3G). The protection of Polβ by its interaction with NQO1/NADH suggests that NQO1 may play a role as a gatekeeper of the 20S proteasome and that NQO1 may prevent Polβ targeted degradation by the 20S proteasome. Overall, this highlights a regulatory role for NQO1 in the ubiquitin-independent degradation of Polβ.

### Oxidative stress promotes the dissociation of Polβ and NQO1 and enhances the association of Polβ with XRCC1

**Figure 4.**
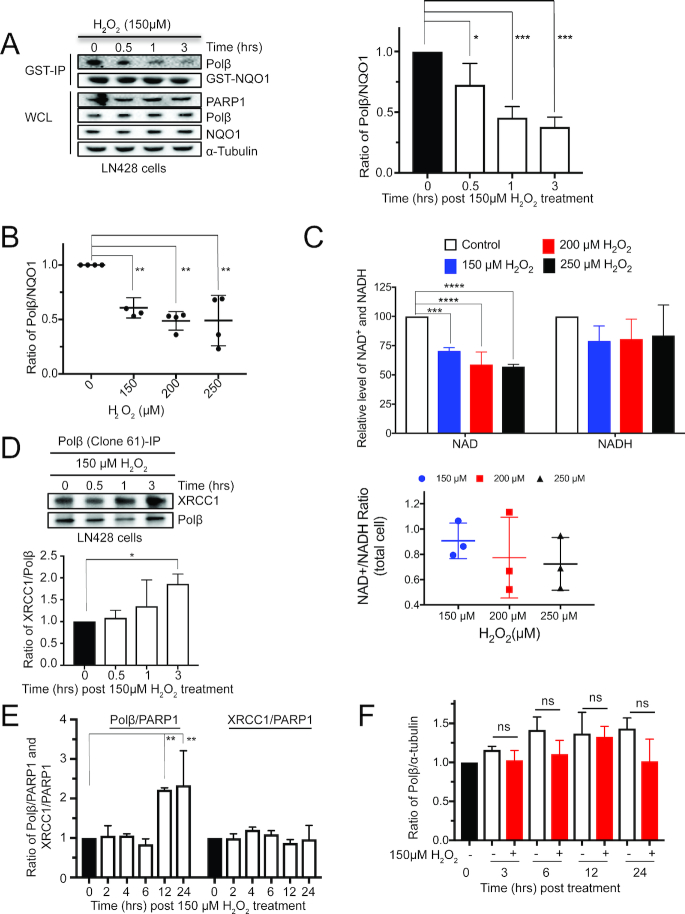
Oxidative stress promotes the dissociation of Polβ with NQO1 and enhances the association of Polβ with XRCC1. (**A**) H_2_O_2_ treatment of LN428 cells promotes the dissociation of the Polβ/NQO1 complex. The representative immunoblot shows the proteins in LN428 cell lysates bound to GST-NQO1, including Polβ, PARP1 and XRCC1, respectively, and the variation of the bound proteins upon treatment of LN428 cells with H_2_O_2_ (150 μM, 0–3 h). The images of two other independent experiments are shown in Supplementary Figure S4A. The level of Polβ and of NQO1 was determined by densitometry and was quantified using Image Lab (Bio-Rad). The ratio of Polβ/NQO1 was calculated and plotted (right panel); * *P* < 0.05, *** *P* < 0.001, compared to time = 0. One-way ANOVA with Dunnett’s multiple comparisons test was used for the plot shown. (**B**) H_2_O_2_ treatment of LN428 cells (dose response) promotes the dissociation of the Polβ/NQO1 complex. The images of four independent immunoblot analyses (Supplementary Figure S4B) show the proteins in LN428 cell lysates bound to GST-NQO1, including Polβ, PARP1 and XRCC1, respectively, and the variation of the bound proteins upon treatment of LN428 cells with H_2_O_2_ (0–250 μM, 3 h). The level of Polβ and NQO1 was determined by densitometry and was quantified using Image Lab (Bio-Rad) and the ratio of Polβ/NQO1 was calculated and plotted; ** *P* < 0.01, comparison to cells without H_2_O_2_ treatment. One-way ANOVA with Dunnett’s multiple comparisons test was used for the plot shown. (**C**) Plot (top) shows the relative level of NAD^+^ and NADH in LN428 cells following treatment with H_2_O_2_ (0, 150, 200 and 250 μM, 3 h); *** *P* < 0.0005, compared to untreated cells; **** *P* < 0.0001, compared to untreated cells. Plot (bottom) indicates the ratio of NAD^+^ to NADH calculated from the plot above. One-way ANOVA with Dunnett’s multiple comparisons test was used for both plots. (**D**) H_2_O_2_ treatment promotes the association of Polβ with XRCC1 in LN428 cells. The representative immunoblot (top panel) shows the proteins immunoprecipitated using an Ab to Polβ (monoclonal Ab, clone 61). Proteins were analyzed from control LN428 cells or those treated with H_2_O_2_ (150 μM, 0.5–3 h). The immunoprecipitated proteins were probed by immunoblot for the level of Polβ and XRCC1. The images of two additional independent experiments are shown in Supplementary Figure S4E. The ratio of XRCC1/Polβ was quantified and plotted; * *P* < 0.05, compared to time = 0. One-way ANOVA with Dunnett’s multiple comparisons test was used for the plot shown. (**E**) Oxidative stress promotes nuclear translocation of Polβ but not XRCC1 in LN428 cells. The sub-cellular distribution of Polβ in LN428 cells, either control cells or those treated with H_2_O_2_ (150 μM, 0–24 h), was evaluated by immunoblot. Proteins of the cytosol and chromatin fractions from LN428 cells were isolated (10 μl of cytosol and chromatin fractions were loaded and the level of Polβ, PARP1, XRCC1 and α-tubulin was examined by immunoblot). The images of three independent experiments are shown in Supplementary Figure S4H. The levels of Polβ, XRCC1 and PARP1 in the chromatin fraction were determined by densitometry and quantified using Image Lab (Bio-Rad). The ratio of Polβ/PARP1 and XRCC1/PARP1 was calculated and plotted; ** *P* < 0.01, compared to time = 0. One-way ANOVA with Dunnett’s multiple comparisons test was used for the plot shown. (**F**) Oxidative stress does not alter the basal expression level of Polβ in LN428 cells. The level of Polβ in WCL in LN428 cells, either control cells or those treated with H_2_O_2_ (150 μM, 0–24 h), was evaluated by immunoblot (25 μg of WCL were loaded and the level of Polβ, PARP1, XRCC1 and α-tubulin was examined by immunoblot). The images of three independent experiments are shown in Supplementary Figure S4J. The levels of Polβ and α-tubulin were quantified using the Image Lab software (Bio-Rad), and the ratio of Polβ/α-tubulin was determined by densitometry and quantified using Image Lab (Right Panel). ns: *P* > 0.05, one-way ANOVA with Dunnett’s multiple comparisons test was used for the plot shown.

The proteolytic capacity of the 20S proteasome is elevated in cells responding to oxidative stress (35,59,60). NQO1, a known regulator of the 20S proteasome, plays an important role in cells exposed to oxidative stress (61,62), and it has been suggested that the interaction of NQO1 with target proteins such as p53 and ODC is modulated by oxidative stress (53,54). Since we found that Polβ interacts with NQO1, we evaluated how oxidative stress affects the Polβ/NQO1 interaction. We find that exposure of LN428 cells to H_2_O_2_ (150 μM, up to 3 h) promotes the dissociation of NQO1 from Polβ in a time-dependent manner, resulting in the loss of >50% of the level of Polβ bound to NQO1 (Figure 4A and Supplementary Figure S4A), with no significant change at increased doses of H_2_O_2_ (Figure 4B and Supplementary Figure S4B). Conversely, the abundance of NQO1, Polβ and other BER-related proteins remain constant (Figure 4A and Supplementary Figure S4A). Similar results were also seen when evaluating the impact of H_2_O_2_ treatment on the Polβ/NQO1 complex in T98G cells (Supplementary Figure S4C and D). Interestingly, the level of NQO1 in T98G cells is highly elevated as compared to LN428 cells (Figure 3E), impacting the oxidative stress-induced effect on the Polβ/NQO1 interaction (Supplementary Figure S4C and D).

Hydrogen peroxide (H_2_O_2_) treatment damages DNA sufficiently to activate PARP1 to produce poly(ADP-ribose), resulting in the cellular depletion of NAD^+^ and ATP (48,63). Since regulation of the 20S proteasome by NQO1 is NADH-dependent (53,54,56), we hypothesized that H_2_O_2_ treatment may therefore alter the NAD^+^/NADH ratio to promote the dissociation of the NQO1/Polβ complex. To that end, we measured the level of NAD^+^ and NADH in LN428 cells exposed to H_2_O_2_ for 3 h (150, 200 and 250 μM). While the treatment significantly decreased the level of NAD^+^, there was no significant change in the level of NADH (Figure 4C), suggesting that it is the level of NAD^+^ or the NAD^+^/NADH ratio that may impact the status of the NQO1/Polβ complex.

H_2_O_2_ treatment results in the oxidation of many proteins (35), potentially altering protein function or protein complex formation. The oxidation of XRCC1 enhances its binding affinity with Polβ by forming additional hydrophobic interactions (36,64) (Figure 1A), thereby promoting Polβ recruitment to sites of DNA damage (65). This would suggest that oxidative stress may trigger a switch, promoting the dissociation of the Polβ/NQO1 complex (Figure 4A and B) and the association of Polβ with the oxidized form of XRCC1. To test this hypothesis, we evaluated the level of Polβ bound XRCC1 by IP/IB following treatment of cells with H_2_O_2_ (150 μM, 0–3 h). In-line with the increased binding affinity of oxidized XRCC1 for Polβ (36,64), we find an increase in the Polβ/XRCC1 complex in response to H_2_O_2_ treatment in LN428 cells (Figure 4D and Supplementary Figure S4E and F) and T98G cells (Supplementary Figure S4G). The increase in the level of the Polβ/XRCC1 complex may be a reflection of either an increase in the level of Polβ localized to chromatin or an increase in Polβ expression. Upon H_2_O_2_ treatment of LN428 cells, we find that there is an increased level of Polβ in the chromatin fraction when normalized to the level of PARP1 at 12 and 24 h post-treatment, whereas there is no change in the XRCC1/PARP1 ratio (Figure 4E and Supplementary Figure S4H and I). However, we find no change in the total level of Polβ in whole cell lysates (Figure 4F and Supplementary Figure S4J and K). To confirm this finding, we used immunofluorescence confocal microscopy to quantify nuclear Polβ and evaluate changes in Polβ staining in response to H_2_O_2_ (150 μM, 24 h). In-line with the biochemical/immunoblot analyses, we find a significant increase in Polβ nuclear staining following H_2_O_2_ treatment (100–200 cells for each condition; ****P < 0.00001 (Supplementary Figure S4L).

### The interaction of Polβ with XRCC1 promotes chromatin localization of Polβ

**Figure 5.**
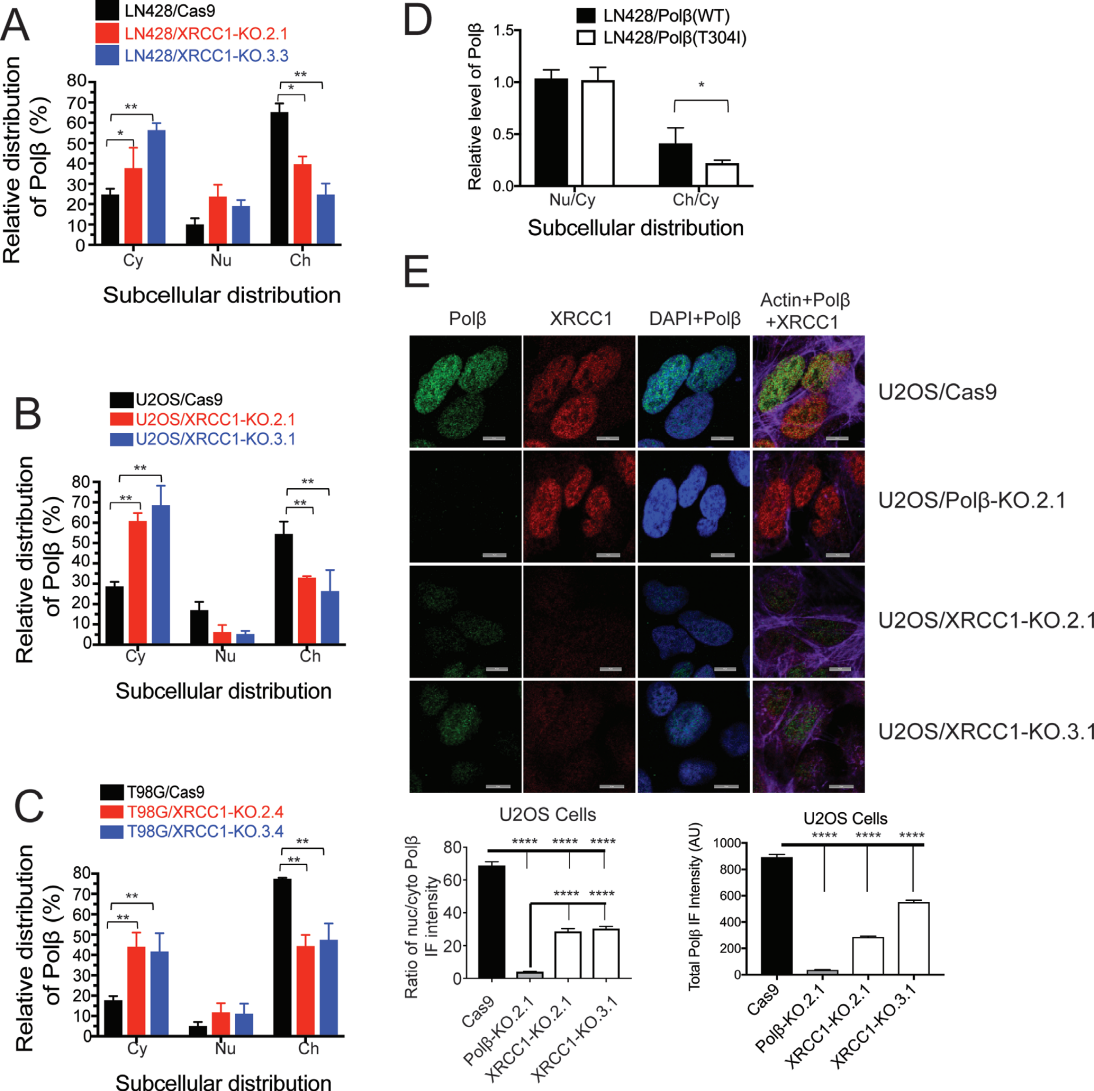
Polβ/XRCC1 interaction promotes nuclear and chromatin localization of Polβ. (**A–C**) CRISPR/Cas9-mediated knockout of XRCC1 reduces chromatin localization of Polβ. Cells were fractionated into cytoplasmic (Cy), nucleoplasmic (Nu) and chromatin (Ch) fractions and probed by immunoblot. Levels of Polβ in each fraction were determined by densitometry and quantified using Image Lab. Relative distribution of Polβ between Cy, Nu and Ch fractions was measured and compared by one-way ANOVA followed by Dunnett’s multiple comparison test. Reduced chromatin localization was observed in LN428 (Panel A, Supplementary Figure S5G), U2OS (Panel B, Supplementary Figure S5D) and T98G (Panel C, Supplementary Figure S5F) cell lines; * *P* < 0.05, ** *P* < 0.01. (**D**) Polβ mutant incapable of binding to XRCC1 exhibits reduced chromatin localization. Flag-Polβ(WT) and Flag-Polβ(T304I) were expressed in U2OS cells and levels of Polβ in each fraction from four immunoblots (Supplementary Figure S5A) were measured. Polβ levels are expressed as ratios relative to cytoplasmic Polβ levels for nucleoplasmic (Nu/Cy) and chromatin (Ch/Cy) fractions, and ratios were compared by Student’s *t*-test; * *P* < 0.05. (**E**) The sub-cellular distribution of Polβ and XRCC1 in U2OS cells was also evaluated by immunofluorescence. Polβ and XRCC1 were probed with anti-Polβ and anti-XRCC1 antibodies, and the nuclear and cytoplasmic compartments were defined by staining with DAPI and phalloidin conjugated to AlexFluor 647, respectively. Top panel: Images were collected (scale bar = 10 μm) and staining intensity was quantified using a custom analysis macro written for NIS-Elements. Bottom panels: Quantified data were compared using one-way ANOVA followed by Tukey’s multiple comparison test. U2OS cells in which XRCC1 was knocked out show a reduction in both overall levels of Polβ (left) and nuclear/cytoplasmic ratios of Polβ (right); **** *P* < 0.0001.

The interaction of Polβ with XRCC1 plays a key role in recruiting Polβ to sites of DNA damage (18,66). As we have shown, mutations in Polβ that block its interaction with XRCC1 reduce the stability of Polβ and diminish its ability to be recruited to sites of DNA damage (18). Given this role of XRCC1 in facilitating the recruitment of Polβ, it is also conceivable that the interaction between Polβ and XRCC1 may dictate the sub-cellular distribution of Polβ. To test this possibility, we generated XRCC1 knockout cell lines using CRISPR/Cas9 technology in LN428, U2OS and T98G cells. We then isolated protein fractions of the cytosol, nucleoplasm and chromatin from these cell lines. Expression of Cas9 had no effect on the level or distribution of Polβ or XRCC1 in U2OS (Supplementary Figure S5B), T98G (Supplementary Figure S5E and F) or LN428 cells (Supplementary Figure S5G). However, loss of XRCC1 caused a significant alteration of the distribution and levels of Polβ in all three cell lines. Relative levels of Polβ in the chromatin fraction were reduced, while levels of Polβ in the cytoplasm were increased in all three knockout cell lines relative to their respective parental cells (Figure 5A–C). Overall, levels of Polβ were also reduced in all three XRCC1-KO cell lines relative to parental control cells (Supplementary Figure S5D–G). These data strongly suggest a role for XRCC1 in the sub-cellular distribution of Polβ as well as the overall level of Polβ, the latter in-line with our earlier report and as reported by others (18,67). To assess whether this is due to transcriptional regulation or due to the disruption of the Polβ/XRCC1 complex, we expressed the Flag-tagged Polβ mutants that are incapable of interacting with XRCC1 as well as Flag-Polβ(WT) in LN428 cells, then probed the isolated protein fractions, as above. The relative levels of Flag-Polβ(T304I) in the chromatin fraction were significantly lower than that of Flag-Polβ(WT) (Figure 5D and Supplementary Figure S5A), strongly suggesting that Polβ’s interaction with XRCC1 facilitates its chromatin localization. To further assess the role of XRCC1 in Polβ distribution and levels, we used immunofluorescence to visualize and quantify relative Polβ levels in the nucleus and cytosol. In agreement with our biochemical data, we observed a shift from nuclear to cytoplasmic localization as well as a reduction in overall levels of Polβ in U2OS/XRCC1-KO (Figure 5E) and T98G/XRCC1-KO (Supplementary Figure S5I) cell lines relative to parental cells. Together, these data provide strong evidence that the interaction between Polβ and XRCC1 is required for Polβ sub-cellular distribution and stability.

## DISCUSSION

DNA repair pathways maintain the integrity of the genome and thereby help prevent the onset of cancer, disease and aging phenotypes (68). Consequently, it has been suggested that all cancer cells are likely defective in some aspect of DNA repair (69,70). Protein–protein interactions are essential for most cellular functions, including but not limited to replication, transcription, mitochondrial function, apoptosis and DNA repair (19,71–74). The BER pathway can be represented as a series of coordinated and sequential DNA repair protein complexes. These repair complexes rely on critical protein–protein interactions to promote assembly in response to post-translational protein modifications to facilitate repair (19). As such, mutations in critical protein complex interfaces could disrupt and inhibit DNA repair.

One of the central and critical protein sub-complexes in BER is the heterodimer of DNA polymerase β (Polβ) and XRCC1. Polβ and XRCC1 form a tight complex via an interaction between the C-terminal domain of Polβ and the N-terminal domain of XRCC1 (21,36,37). As we have reported, the Polβ/XRCC1 heterodimer plays an important role in regulating the ubiquitylation and degradation of Polβ (18). A TCGA analysis of the majority of the BER genes reveals they are altered in 3977 (9%) of 44648 sequenced cases/patients (46 139 total) (75,76). Missense, frame shift and truncating mutations for Polβ and XRCC1 are uniformly found across the gene for each (Supplementary Figure S6). Interestingly, among the Pan Cancer TCGA samples, only three missense mutations were found in XRCC1 within the Polβ-interaction domain (spanning residues 67–86; A79T, E81D and E85K), while no missense mutations were identified in Polβ within the XRCC1-interaction domain (spanning residues 301–309). A more detailed analysis of mutations that may impact protein complex formation has yet to be accomplished.

Of the many mutations recently identified in a large cohort of colon cancer patients, as many as 75% of the tumors presented with *POLB* gene mutations (11). One of these colon cancer mutations is located within the XRCC1-interacting domain or V303 loop, the T304I mutation (11). In our investigation of the cellular impact of the T304I cancer mutation of DNA Polymerase β (Polβ), we find that mutation of this surface threonine residue impacts critical and novel Polβ protein–protein interactions. In-line with our earlier report (18), the T304I mutation, found within the V303 loop, significantly impacts the ability of Polβ to interact with XRCC1. As we show herein, we observe reduced interaction between Polβ(T304I) and XRCC1 in cell lysates and a significant decrease in binding affinity when evaluating purified proteins (the binding affinity of Polβ(WT) to XRCC1 is 2.38- to 12.48-fold higher than that of Polβ(T304I)). Also predicted from our previous report (18) is the instability of the Polβ(T304I) protein that is triggered by the reduced binding affinity for XRCC1. In addition, the reduced interaction of the Polβ(T304I) mutant protein with XRCC1 revealed a novel Polβ interaction partner protein, NAD(P)H quinone dehydrogenase 1 (NQO1), a known regulator of the 20S proteasome. Overall, we find that the proteasome-mediated degradation of Polβ is regulated by both ubiquitin-dependent and ubiquitin-independent processes.

The present study highlights a novel mechanism of a ubiquitin-independent proteasome pathway regulating the level of Polβ, whereby NQO1 interacts with and stabilizes Polβ by preventing its degradation in the cytosol. We demonstrate here that modification of the *bona fide* Polβ ubiquitylation site residues K206 and K244 (18) completely blocks the ubiquitylation of Polβ(T304I) yet fails to stabilize the Polβ(T304I) protein and in fact promotes its degradation. Together, we find that the ubiquitin-independent proteasome pathway regulates the stability of Polβ in the cytosol, via an interaction between Polβ and NQO1 in an NADH-dependent manner. Conversely, the interaction of Polβ with XRCC1 plays a key role regulating the stability of Polβ via a ubiquitin-dependent pathway.

The 20S proteasome preferentially degrades oxidized proteins (35,77) and in many cases, the capacity of the 20S proteasome is elevated in cells responding to oxidative stress (35,59,60). Proteins known to regulate the function of the 20S proteasome include NQO1 (35). Oxidative stress promotes the association of NQO1 with p53 yet mediates the dissociation of NQO1 from ODC (53,54). Given the critical role for the BER pathway in the cellular response to oxidative stress-induced DNA damage, it is interesting that we find these two pathways converge. Here, we show that oxidative stress promotes the dissociation of the Polβ/NQO1 complex, enhancing the interaction of Polβ with XRCC1. Interestingly, the basal level of Polβ, XRCC1 and NQO1 proteins do not change in response to oxidative stress, suggesting that the interaction of Polβ with XRCC1 plays a key role in stabilizing Polβ when dissociated from NQO1. Oxidative stress impacted the level of NAD^+^ rapidly and had a minor impact (if any) on the level of cellular NADH. Although we observe only a minimal change in NADH, the overall NAD^+^/NADH ratio is reduced. This is similar to what we have found for treatment of cells with alkylating agents, activating a strong PARP1 response, a reduction in NAD^+^ yet no measurable reduction in NADH (48). It is likely that the local concentration of NADH (cytosolic) is altered to a greater extent, promoting the dissociation of Polβ from NQO1. However, it should be noted that the ratio of free NADH/NAD^+^ is ∼0.0001 in the cytosol, while it is ∼0.143 in the mitochondria (78). As such, the bound fraction of NADH dominates the measurement (79), thereby limiting the measurable change in free NADH upon oxidative stress, especially within a few hours of exposure. This would be consistent with known sub-cellular changes in NAD^+^ and NAD-metabolites in response to cellular stress (48,80).

Efficient BER requires localization of Polβ in the nucleus and, more specifically, in the chromatin fraction so as to be available for repair of base damage (81). As with many proteins, Polβ contains a nuclear localization sequence, located at its N-terminus, contributing to the transport of Polβ to the nucleus (82). Herein, we find that XRCC1 also influences the nuclear localization of Polβ, potentially impacting the capacity of Polβ for recruitment to sites of DNA damage. In the absence of XRCC1, Polβ protein is reduced in both the nuclear and chromatin protein fraction and by immunofluorescence confocal microscopy, we find a reduction in the total level of Polβ and a reduction in the nuclear-to-cytosolic ratio of Polβ.

**Figure 6.**
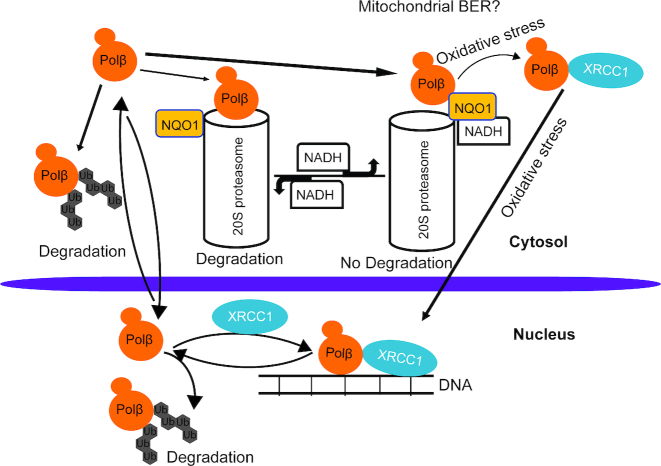
Oxidative stress-induced regulation of Polβ protein sub-cellular localization. Proposed model highlighting the regulation of Polβ protein sub-cellular localization by oxidative stress. The Polβ/NQO1 interaction regulates the level of Polβ in the cytosol by preventing the ubiquitin-independent proteasome pathway. Conversely, the Polβ/XRCC1 interaction regulates the level of Polβ in the nucleus by preventing activation of the ubiquitin-dependent proteasome pathway. Oxidative stress promotes the regulation of the binding partners, alternating from Polβ bound to NQO1 ⇒ Polβ bound to XRCC1, promoting the chromatin localization of Polβ.

In this study, we have characterized the colon cancer mutant of Polβ, T304I. Our analysis has revealed a new model that may govern Polβ protein stability and sub-cellular localization (Figure 6). Specifically, we find that the degradation of Polβ is regulated by both ubiquitin-dependent and ubiquitin-independent proteasome processes. In this context, Polβ forms two unique protein complexes. The interaction with NQO1 regulates the stability of Polβ in the cytosol. Given the recent discovery that Polβ plays a role in mitochondrial BER by us and others (83,84), maintaining the cytosolic stability of Polβ may be important for mitochondrial genome maintenance. Conversely, we find that oxidative stress promotes the dissociation of the Polβ/NQO1 complex, enhancing the interaction of Polβ with XRCC1. The interaction with XRCC1 then regulates the stability of Polβ via a ubiquitin-dependent pathway. Further, we find that XRCC1 plays a key role in the sub-cellular localization of Polβ that is expected to impact the ability of Polβ to be recruited to sites of DNA damage. Overall, our results reveal that somatic mutations that mitigate protein–protein interactions or disrupt key protein complexes can impact protein function by regulating protein stability and sub-cellular localization.

## Supplementary Material

gkz293_Supplemental_Files

## References

[1] Jeggo P.A., Pearl L.H., Carr A.M. DNA repair, genome stability and cancer: a historical perspective. Nat. Rev. Cancer. 2016; 16:35–42. 26667849 10.1038/nrc.2015.4

[2] Laverty D.J., Mortimer I.P., Greenberg M.M. Mechanistic insight through irreversible inhibition: DNA polymerase theta uses a common active site for polymerase and lyase activities. J. Am. Chem. Soc. 2018; 140:9034–9037. 29998737 10.1021/jacs.8b04158 PMC6085753

[3] Roth M., Wang Z., Chen W.Y. SIRT1 and LSD1 competitively regulate KU70 functions in DNA repair and mutation acquisition in cancer cells. Oncotarget. 2016; 7:50195–50214. 27384990 10.18632/oncotarget.10328 PMC5226577

[4] Rizzo A.A., Vassel F.M., Chatterjee N., D'Souza S., Li Y., Hao B., Hemann M.T., Walker G.C., Korzhnev D.M. Rev7 dimerization is important for assembly and function of the Rev1/Polzeta translesion synthesis complex. Proc. Natl. Acad. Sci. U.S.A. 2018; 115:E8191–E8200. 30111544 10.1073/pnas.1801149115 PMC6126741

[5] Coulombe B., Jeronimo C., Langelier M.F., Cojocaru M., Bergeron D. Interaction networks of the molecular machines that decode, replicate, and maintain the integrity of the human genome. Mol. Cell Proteom. 2004; 3:851–856. 10.1074/mcp.R400009-MCP200 PMC4494826 15215308

[6] Barnes D.E., Lindahl T. Repair and genetic consequences of endogenous DNA base damage in mammalian cells. Annu. Rev. Genet. 2004; 38:445–476. 15568983 10.1146/annurev.genet.38.072902.092448

[7] Sobol R.W., Horton J.K., Kuhn R., Gu H., Singhal R.K., Prasad R., Rajewsky K., Wilson S.H. Requirement of mammalian DNA polymerase-beta in base-excision repair. Nature. 1996; 379:183–186. 8538772 10.1038/379183a0

[8] Sobol R.W., Prasad R., Evenski A., Baker A., Yang X.P., Horton J.K., Wilson S.H. The lyase activity of the DNA repair protein beta-polymerase protects from DNA-damage-induced cytotoxicity. Nature. 2000; 405:807–810. 10866204 10.1038/35015598

[9] Starcevic D., Dalal S., Sweasy J.B. Is there a link between DNA polymerase beta and cancer. Cell Cycle. 2004; 3:998–1001. 15280658

[10] Tan X.H., Zhao M., Pan K.F., Dong Y., Dong B., Feng G.J., Jia G., Lu Y.Y. Frequent mutation related with overexpression of DNA polymerase beta in primary tumors and precancerous lesions of human stomach. Cancer Lett. 2005; 220:101–114. 15737693 10.1016/j.canlet.2004.07.049

[11] Donigan K.A., Sun K.W., Nemec A.A., Murphy D.L., Cong X., Northrup V., Zelterman D., Sweasy J.B. Human POLB gene is mutated in high percentage of colorectal tumors. J. Biol. Chem. 2012; 287:23830–23839. 22577134 10.1074/jbc.M111.324947 PMC3390656

[12] Nemec A.A., Wallace S.S., Sweasy J.B. Variant base excision repair proteins: contributors to genomic instability. Semin. Cancer Biol. 2010; 20:320–328. 20955798 10.1016/j.semcancer.2010.10.010 PMC3254599

[13] Eckenroth B.E., Towle-Weicksel J.B., Nemec A.A., Murphy D.L., Sweasy J.B., Doublie S. Remote Mutations Induce Functional Changes in Active Site Residues of Human DNA Polymerase beta. Biochemistry. 2017; 56:2363–2371. 28402631 10.1021/acs.biochem.6b01287 PMC5501977

[14] Guo Z., Zheng L., Dai H., Zhou M., Xu H., Shen B. Human DNA polymerase beta polymorphism, Arg137Gln, impairs its polymerase activity and interaction with PCNA and the cellular base excision repair capacity. Nucleic Acids Res. 2009; 37:3431–3441. 19336415 10.1093/nar/gkp201 PMC2691839

[15] Yamtich J., Nemec A.A., Keh A., Sweasy J.B. A germline polymorphism of DNA polymerase beta induces genomic instability and cellular transformation. PLoS Genet. 2012; 8:e1003052. 23144635 10.1371/journal.pgen.1003052 PMC3493456

[16] Dianov G.L., Prasad R., Wilson S.H., Bohr V.A. Role of DNA polymerase beta in the excision step of long patch mammalian base excision repair. J. Biol. Chem. 1999; 274:13741–13743. 10318775 10.1074/jbc.274.20.13741

[17] Beard W.A., Wilson S.H. Structure and mechanism of DNA polymerase Beta. Chem. Rev. 2006; 106:361–382. 16464010 10.1021/cr0404904

[18] Fang Q., Inanc B., Schamus S., Wang X.H., Wei L., Brown A.R., Svilar D., Sugrue K.F., Goellner E.M., Zeng X. et al. HSP90 regulates DNA repair via the interaction between XRCC1 and DNA polymerase beta. Nat. Commun. 2014; 5:5513. 25423885 10.1038/ncomms6513 PMC4246423

[19] Almeida K.H., Sobol R.W. A unified view of base excision repair: lesion-dependent protein complexes regulated by post-translational modification. DNA Repair. 2007; 6:695–711. 17337257 10.1016/j.dnarep.2007.01.009 PMC1995033

[20] Wilson S.H., Kunkel T.A. Passing the baton in base excision repair. Nat. Struct. Biol. 2000; 7:176–178. 10700268 10.1038/73260

[21] Marintchev A., Robertson A., Dimitriadis E.K., Prasad R., Wilson S.H., Mullen G.P. Domain specific interaction in the XRCC1-DNA polymerase beta complex. Nucleic Acids Res. 2000; 28:2049–2059. 10773072 10.1093/nar/28.10.2049 PMC105377

[22] Gryk M.R., Marintchev A., Maciejewski M.W., Robertson A., Wilson S.H., Mullen G.P. Mapping of the interaction interface of DNA polymerase beta with XRCC1. Structure. 2002; 10:1709–1720. 12467578 10.1016/s0969-2126(02)00908-5

[23] Prasad R., Dianov G.L., Bohr V.A., Wilson S.H. FEN1 stimulation of DNA polymerase beta mediates an excision step in mammalian long patch base excision repair. J. Biol. Chem. 2000; 275:4460–4466. 10660619 10.1074/jbc.275.6.4460

[24] Prasad R., Liu Y., Deterding L.J., Poltoratsky V.P., Kedar P.S., Horton J.K., Kanno S., Asagoshi K., Hou E.W., Khodyreva S.N. et al. HMGB1 is a cofactor in mammalian base excision repair. Mol. Cell. 2007; 27:829–841. 17803946 10.1016/j.molcel.2007.06.029 PMC2799894

[25] Bennett R.A.O., Wilson D.M. 3rd, Wong D., Demple B. Interaction of human apurinic endonuclease and DNA polymerase ß in the base excision repair pathway. Proc. Natl. Acad. Sci. U.S.A. 1997; 94:7166–7169. 9207062 10.1073/pnas.94.14.7166 PMC23779

[26] Kedar P.S., Kim S.J., Robertson A., Hou E., Prasad R., Horton J.K., Wilson S.H. Direct interaction between mammalian DNA polymerase beta and proliferating cell nuclear antigen. J. Biol. Chem. 2002; 277:31115–31123. 12063248 10.1074/jbc.M201497200

[27] Zhou J., Ahn J., Wilson S.H., Prives C. A role for p53 in base excision repair. EMBO J. 2001; 20:914–923. 11179235 10.1093/emboj/20.4.914 PMC145418

[28] Cabelof D.C., Guo Z., Raffoul J.J., Sobol R.W., Wilson S.H., Richardson A., Heydari A.R. Base excision repair deficiency caused by polymerase beta haploinsufficiency: accelerated DNA damage and increased mutational response to carcinogens. Cancer Res. 2003; 63:5799–5807. 14522902

[29] Cabelof D.C., Ikeno Y., Nyska A., Busuttil R.A., Anyangwe N., Vijg J., Matherly L.H., Tucker J.D., Wilson S.H., Richardson A. et al. Haploinsufficiency in DNA polymerase beta increases cancer risk with age and alters mortality rate. Cancer Res. 2006; 66:7460–7465. 16885342 10.1158/0008-5472.CAN-06-1177

[30] Canitrot Y., Cazaux C., Frechet M., Bouayadi K., Lesca C., Salles B., Hoffmann J.S. Overexpression of DNA polymerase beta in cell results in a mutator phenotype and a decreased sensitivity to anticancer drugs. Proc. Natl. Acad. Sci. U.S.A. 1998; 95:12586–12590. 9770529 10.1073/pnas.95.21.12586 PMC22874

[31] Bergoglio V., Pillaire M.J., Lacroix-Triki M., Raynaud-Messina B., Canitrot Y., Bieth A., Gares M., Wright M., Delsol G., Loeb L.A. et al. Deregulated DNA polymerase beta induces chromosome instability and tumorigenesis. Cancer Res. 2002; 62:3511–3514. 12067997

[32] Yoshizawa K., Jelezcova E., Brown A.R., Foley J.F., Nyska A., Cui X., Hofseth L.J., Maronpot R.M., Wilson S.H., Sepulveda A.R. et al. Gastrointestinal hyperplasia with altered expression of DNA polymerase beta. PLoS One. 2009; 4:e6493. 19654874 10.1371/journal.pone.0006493 PMC2716528

[33] Roos W.P., Thomas A.D., Kaina B. DNA damage and the balance between survival and death in cancer biology. Nat. Rev. Cancer. 2016; 16:20–33. 26678314 10.1038/nrc.2015.2

[34] Thompson L.H. Recognition, signaling, and repair of DNA double-strand breaks produced by ionizing radiation in mammalian cells: the molecular choreography. Mutat. Res. 2012; 751:158–246. 22743550 10.1016/j.mrrev.2012.06.002

[35] Ben-Nissan G., Sharon M. Regulating the 20S proteasome ubiquitin-independent degradation pathway. Biomolecules. 2014; 4:862–884. 25250704 10.3390/biom4030862 PMC4192676

[36] Cuneo M.J., London R.E. Oxidation state of the XRCC1 N-terminal domain regulates DNA polymerase beta binding affinity. Proc. Natl. Acad. Sci. U.S.A. 2010; 107:6805–6810. 20351257 10.1073/pnas.0914077107 PMC2872404

[37] Marintchev A., Mullen M.A., Maciejewski M.W., Pan B., Gryk M.R., Mullen G.P. Solution structure of the single-strand break repair protein XRCC1 N-terminal domain. Nat. Struct. Biol. 1999; 6:884–893. 10467102 10.1038/12347

[38] Trivedi R.N., Wang X.H., Jelezcova E., Goellner E.M., Tang J.B., Sobol R.W. Human methyl purine DNA glycosylase and DNA polymerase beta expression collectively predict sensitivity to temozolomide. Mol. Pharmacol. 2008; 74:505–516. 18477668 10.1124/mol.108.045112 PMC3909956

[39] Tang J.B., Goellner E.M., Wang X.H., Trivedi R.N., St Croix C.M., Jelezcova E., Svilar D., Brown A.R., Sobol R.W. Bioenergetic metabolites regulate base excision repair-dependent cell death in response to DNA damage. Mol. Cancer Res. 2010; 8:67–79. 20068071 10.1158/1541-7786.MCR-09-0411 PMC2808464

[40] Beard W.A., Wilson S.H. Purification and domain-mapping of mammalian DNA polymerase beta. Methods Enzymol. 1995; 262:98–107. 8594388 10.1016/0076-6879(95)62013-3

[41] Freudenthal B.D., Beard W.A., Shock D.D., Wilson S.H. Observing a DNA polymerase choose right from wrong. Cell. 2013; 154:157–168. 23827680 10.1016/j.cell.2013.05.048 PMC3924593

[42] Willets K.A., Van Duyne R.P. Localized surface plasmon resonance spectroscopy and sensing. Annu. Rev. Phys. Chem. 2007; 58:267–297. 17067281 10.1146/annurev.physchem.58.032806.104607

[43] Svilar D., Dyavaiah M., Brown A.R., Tang J.B., Li J., McDonald P.R., Shun T.Y., Braganza A., Wang X.H., Maniar S. et al. Alkylation sensitivity screens reveal a conserved cross-species functionome. Mol. Cancer Res. 2012; 10:1580–1596. 23038810 10.1158/1541-7786.MCR-12-0168 PMC3877719

[44] Hsu P.D., Scott D.A., Weinstein J.A., Ran F.A., Konermann S., Agarwala V., Li Y., Fine E.J., Wu X., Shalem O. et al. DNA targeting specificity of RNA-guided Cas9 nucleases. Nat. Biotechnol. 2013; 31:827–832. 23873081 10.1038/nbt.2647 PMC3969858

[45] Slyskova J., Sabatella M., Ribeiro-Silva C., Stok C., Theil A.F., Vermeulen W., Lans H. Base and nucleotide excision repair facilitate resolution of platinum drugs-induced transcription blockage. Nucleic Acids Res. 2018; 46:9537–9549. 30137419 10.1093/nar/gky764 PMC6182164

[46] Sanjana N.E., Shalem O., Zhang F. Improved vectors and genome-wide libraries for CRISPR screening. Nat. Methods. 2014; 11:783–784. 25075903 10.1038/nmeth.3047 PMC4486245

[47] Shalem O., Sanjana N.E., Hartenian E., Shi X., Scott D.A., Mikkelson T., Heckl D., Ebert B.L., Root D.E., Doench J.G. et al. Genome-scale CRISPR-Cas9 knockout screening in human cells. Science. 2014; 343:84–87. 24336571 10.1126/science.1247005 PMC4089965

[48] Fouquerel E., Goellner E.M., Yu Z., Gagne J.P., Barbi de Moura M., Feinstein T., Wheeler D., Redpath P., Li J., Romero G. et al. ARTD1/PARP1 negatively regulates glycolysis by inhibiting hexokinase 1 independent of NAD+ depletion. Cell Rep. 2014; 8:1819–1831. 25220464 10.1016/j.celrep.2014.08.036 PMC4177344

[49] Wang L., Patel U., Ghosh L., Banerjee S. DNA polymerase beta mutations in human colorectal cancer. Cancer Res. 1992; 52:4824–4827. 1511447

[50] Matsuzaki J., Dobashi Y., Miyamoto H., Ikeda I., Fujinami K., Shuin T., Kubota Y. DNA polymerase beta gene mutations in human bladder cancer. Mol. Carcinog. 1996; 15:38–43. 8561864 10.1002/(SICI)1098-2744(199601)15:1<38::AID-MC6>3.0.CO;2-O

[51] Dobashi Y., Shuin T., Tsuruga H., Uemura H., Torigoe S., Kubota Y. DNA polymerase beta gene mutation in human prostate cancer. Cancer Res. 1994; 54:2827–2829. 8187060

[52] Odell I.D., Barbour J.E., Murphy D.L., Della-Maria J.A., Sweasy J.B., Tomkinson A.E., Wallace S.S., Pederson D.S. Nucleosome disruption by DNA ligase III-XRCC1 promotes efficient base excision repair. Mol. Cell Biol. 2011; 31:4623–4632. 21930793 10.1128/MCB.05715-11 PMC3209256

[53] Asher G., Bercovich Z., Tsvetkov P., Shaul Y., Kahana C. 20S proteasomal degradation of ornithine decarboxylase is regulated by NQO1. Mol. Cell. 2005; 17:645–655. 15749015 10.1016/j.molcel.2005.01.020

[54] Asher G., Tsvetkov P., Kahana C., Shaul Y. A mechanism of ubiquitin-independent proteasomal degradation of the tumor suppressors p53 and p73. Genes Dev. 2005; 19:316–321. 15687255 10.1101/gad.319905 PMC546509

[55] Oh E.T., Kim J.W., Kim J.M., Kim S.J., Lee J.S., Hong S.S., Goodwin J., Ruthenborg R.J., Jung M.G., Lee H.J. et al. NQO1 inhibits proteasome-mediated degradation of HIF-1alpha. Nat. Commun. 2016; 7:13593. 27966538 10.1038/ncomms13593 PMC5171868

[56] Tsvetkov P., Reuven N., Shaul Y. Ubiquitin-independent p53 proteasomal degradation. Cell Death Differ. 2010; 17:103–108. 19557012 10.1038/cdd.2009.67

[57] Hosoda S., Nakamura W., Hayashi K. Properties and reaction mechanism of DT diaphorase from rat liver. J. Biol. Chem. 1974; 249:6416–6423. 4138437

[58] Ma Q., Cui K., Xiao F., Lu A.Y., Yang C.S. Identification of a glycine-rich sequence as an NAD(P)H-binding site and tyrosine 128 as a dicumarol-binding site in rat liver NAD(P)H:quinone oxidoreductase by site-directed mutagenesis. J. Biol. Chem. 1992; 267:22298–22304. 1385397

[59] Grune T., Catalgol B., Licht A., Ermak G., Pickering A.M., Ngo J.K., Davies K.J. HSP70 mediates dissociation and reassociation of the 26S proteasome during adaptation to oxidative stress. Free Radic. Biol. Med. 2011; 51:1355–1364. 21767633 10.1016/j.freeradbiomed.2011.06.015 PMC3172204

[60] Wang X., Yen J., Kaiser P., Huang L. Regulation of the 26S proteasome complex during oxidative stress. Sci. Signal. 2010; 3:ra88. 21139140 10.1126/scisignal.2001232 PMC3140957

[61] Ross D., Siegel D. Functions of NQO1 in cellular protection and CoQ10 metabolism and its potential role as a redox sensitive molecular switch. Front. Physiol. 2017; 8:595. 28883796 10.3389/fphys.2017.00595 PMC5573868

[62] Oh E.T., Park H.J. Implications of NQO1 in cancer therapy. BMB Rep. 2015; 48:609–617. 26424559 10.5483/BMBRep.2015.48.11.190 PMC4911202

[63] Fouquerel E., Sobol R.W. ARTD1 (PARP1) activation and NAD(+) in DNA repair and cell death. DNA Repair. 2014; 23:27–32. 25283336 10.1016/j.dnarep.2014.09.004 PMC4252787

[64] Horton J.K., Stefanick D.F., Gassman N.R., Williams J.G., Gabel S.A., Cuneo M.J., Prasad R., Kedar P.S., Derose E.F., Hou E.W. et al. Preventing oxidation of cellular XRCC1 affects PARP-mediated DNA damage responses. DNA Repair. 2013; 12:774–785. 23871146 10.1016/j.dnarep.2013.06.004 PMC3924596

[65] Horton J.K., Seddon H.J., Zhao M.L., Gassman N.R., Janoshazi A.K., Stefanick D.F., Wilson S.H. Role of the oxidized form of XRCC1 in protection against extreme oxidative stress. Free Radic. Biol. Med. 2017; 107:292–300. 28179111 10.1016/j.freeradbiomed.2017.02.005 PMC5457714

[66] Lan L., Nakajima S., Oohata Y., Takao M., Okano S., Masutani M., Wilson S.H., Yasui A. In situ analysis of repair processes for oxidative DNA damage in mammalian cells. Proc. Natl. Acad. Sci. U.S.A. 2004; 101:13738–13743. 15365186 10.1073/pnas.0406048101 PMC518826

[67] Parsons J.L., Tait P.S., Finch D., Dianova II, Allinson S.L., Dianov G.L. CHIP-mediated degradation and DNA damage-dependent stabilization regulate base excision repair proteins. Mol. Cell. 2008; 29:477–487. 18313385 10.1016/j.molcel.2007.12.027

[68] Friedberg E.C., Walker G.C., Siede W., Wood R.D., Schultz R.A., Ellenberger T. DNA Repair and Mutagenesis. 2006; 2nd edn. Washington, D.C ASM Press.

[69] Alberts B. Redefining cancer research. Science. 2009; 325:1319. 19745119 10.1126/science.1181224

[70] Vens C., Sobol R.W. Johnson DE Cell Death Signaling in Cancer Biology And Treatment. 2013; NY Springer.

[71] Dar K.B., Bhat A.H., Amin S., Anjum S., Reshi B.A., Zargar M.A., Masood A., Ganie S.A. Exploring proteomic drug targets, therapeutic strategies and protein-protein interactions in cancer: mechanistic view. Curr. Cancer Drug Targets. 2018; doi:10.2174/1568009618666180803104631. 10.2174/1568009618666180803104631 30073927

[72] Kang S., Kang M.S., Ryu E., Myung K. Eukaryotic DNA replication: orchestrated action of multi-subunit protein complexes. Mutat. Res. 2018; 809:58–69. 28501329 10.1016/j.mrfmmm.2017.04.002

[73] Lambert M., Jambon S., Depauw S., David-Cordonnier M.H. Targeting transcription factors for cancer treatment. Molecules. 2018; 23:E1479. 29921764 10.3390/molecules23061479 PMC6100431

[74] Roy M.J., Vom A., Czabotar P.E., Lessene G. Cell death and the mitochondria: therapeutic targeting of the BCL-2 family-driven pathway. Br. J. Pharmacol. 2014; 171:1973–1987. 24117105 10.1111/bph.12431 PMC3976616

[75] Gao J., Aksoy B.A., Dogrusoz U., Dresdner G., Gross B., Sumer S.O., Sun Y., Jacobsen A., Sinha R., Larsson E. et al. Integrative analysis of complex cancer genomics and clinical profiles using the cBioPortal. Sci. Signal. 2013; 6:pl1. 23550210 10.1126/scisignal.2004088 PMC4160307

[76] Cerami E., Gao J., Dogrusoz U., Gross B.E., Sumer S.O., Aksoy B.A., Jacobsen A., Byrne C.J., Heuer M.L., Larsson E. et al. The cBio cancer genomics portal: an open platform for exploring multidimensional cancer genomics data. Cancer Discover. 2012; 2:401–404. 10.1158/2159-8290.CD-12-0095 PMC3956037 22588877

[77] Baumeister W., Walz J., Zuhl F., Seemuller E. The proteasome: paradigm of a self-compartmentalizing protease. Cell. 1998; 92:367–380. 9476896 10.1016/s0092-8674(00)80929-0

[78] Nuutinen E.M., Hiltunen J.K., Hassinen I.E. The glutamate dehydrogenase system and the redox state of mitochondrial free nicotinamide adenine dinucleotide in myocardium. FEBS Lett. 1981; 128:356–360. 7262326 10.1016/0014-5793(81)80116-0

[79] Bucher T., Brauser B., Conze A., Klein F., Langguth O., Sies H. State of oxidation-reduction and state of binding in the cytosolic NADH-system as disclosed by equilibration with extracellular lactate-pyruvate in hemoglobin-free perfused rat liver. Eur. J. Biochem. 1972; 27:301–317. 4340564 10.1111/j.1432-1033.1972.tb01840.x

[80] Yang H., Yang T., Baur J.A., Perez E., Matsui T., Carmona J.J., Lamming D.W., Souza-Pinto N.C., Bohr V.A., Rosenzweig A. et al. Nutrient-sensitive mitochondrial NAD+ levels dictate cell survival. Cell. 2007; 130:1095–1107. 17889652 10.1016/j.cell.2007.07.035 PMC3366687

[81] Menoni H., Di Mascio P., Cadet J., Dimitrov S., Angelov D. Chromatin associated mechanisms in base excision repair - nucleosome remodeling and DNA transcription, two key players. Free Radic. Biol. Med. 2017; 107:159–169. 28011149 10.1016/j.freeradbiomed.2016.12.026

[82] Kirby T.W., Gassman N.R., Smith C.E., Zhao M.L., Horton J.K., Wilson S.H., London R.E. DNA polymerase beta contains a functional nuclear localization signal at its N-terminus. Nucleic Acids Res. 2017; 45:1958–1970. 27956495 10.1093/nar/gkw1257 PMC5389473

[83] Prasad R., Caglayan M., Dai D.P., Nadalutti C.A., Zhao M.L., Gassman N.R., Janoshazi A.K., Stefanick D.F., Horton J.K., Krasich R. et al. DNA polymerase beta: A missing link of the base excision repair machinery in mammalian mitochondria. DNA Repair (Amst). 2017; 60:77–88. 29100041 10.1016/j.dnarep.2017.10.011 PMC5919216

[84] Sykora P., Kanno S., Akbari M., Kulikowicz T., Baptiste B.A., Leandro G.S., Lu H., Tian J., May A., Becker K.A. et al. DNA polymerase beta participates in mitochondrial DNA repair. Mol. Cell. Biol. 2017; 37:e00237-17. 28559431 10.1128/MCB.00237-17 PMC5533889

