# Supplementary material for: Stability and sub-cellular localization of DNA polymerase β is regulated by interactions with NQO1 and XRCC1 in response to oxidative stress
Source: Nucleic Acids Res. 2019 Apr 26;47(12):6269–86. doi: 10.1093/nar/gkz293 (PMC6614843; doi:10.1093/nar/gkz293)
Supplement: gkz293_Supplemental_Files [file gkz293_supplemental_files.zip › Supplementary Material.pdf]

Stability and subcellular localization of DNA polymerase β is regulated by interactions with NQO1 and XRCC1 in response to oxidative stress

Qingming Fang, Joel Andrews, Nidhi Sharma, Anna Wilk, Jennifer Clark, Jana Slyskova, Christopher A. Koczor, Hannes Lans, Aishwarya Prakash and Robert W. Sobol*

Supplemental Material

Supplementary Figures S1-S6 and the corresponding figure legends

Supplementary Table S1-S5

References Cited

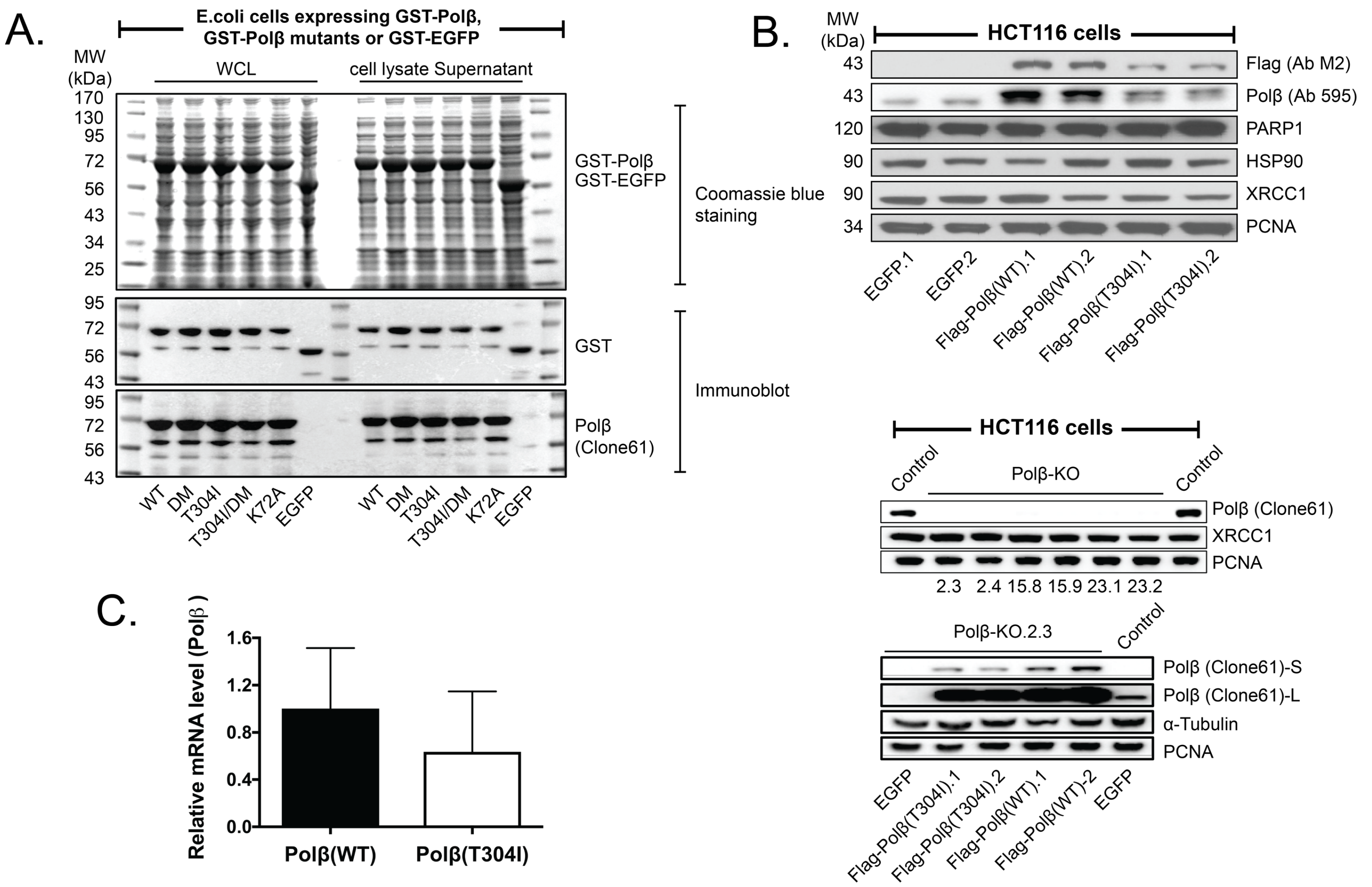

Supplementary Figure S1 (A-C).

(A) The expression profile of GST-Polβ(WT), GST-Polβ mutants (DM, T304**I**, T304**I**/DM and K72A) and GST-EGFP in *E.coli* cells was examined by Coomassie blue staining and immunoblotting. Molecular weights, as determined by marker proteins, are indicated on the left. Antibodies used for immunoblot are indicated on the right.

(B) Top panel: The expression profile of BER related proteins in HCT116 cells modified to express EGFP, Flag-Polβ(WT) or Flag-Polβ(T304**I**) (2 clones each). Molecular weights, as determined by marker proteins, are indicated on the left. Middle panel: A null mutation was engineered in the POLB gene in HCT116 cells using CRISPR/cas9 system (gRNA clone cr2) , as described in Materials and Methods section. Shown is an immunoblot of clones isolated (clones 2.3, 2.4, 15.8, 15.9, 23.1 and 23.2) and two controls. Bottom panel: The expression level of Flag-Polβ(WT), Flag-Polβ(T304**I**) and EGFP in the HCT116/Polβ-KO cells (clone 2.3) was examined by immunoblot. All panels: Antibodies used for immunoblot are indicated on the right; S = short exposure and L = long exposure.

(C) The relative mRNA level of Polβ in HCT116 cells expressing Flag-Polβ(WT) or Flag-Polβ(T304**I**). Comparison by unpaired t-test; not significant.

D.

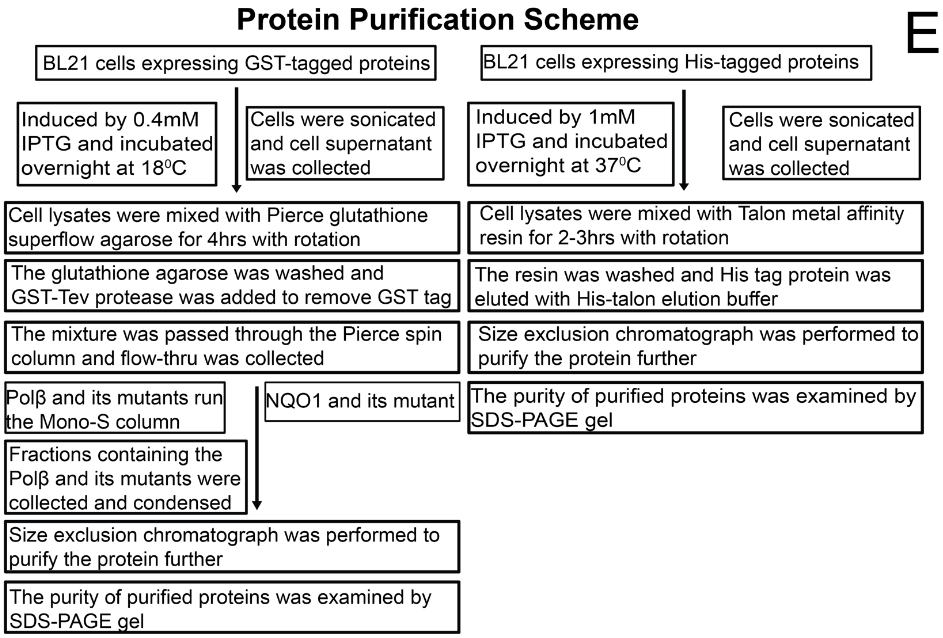

E.

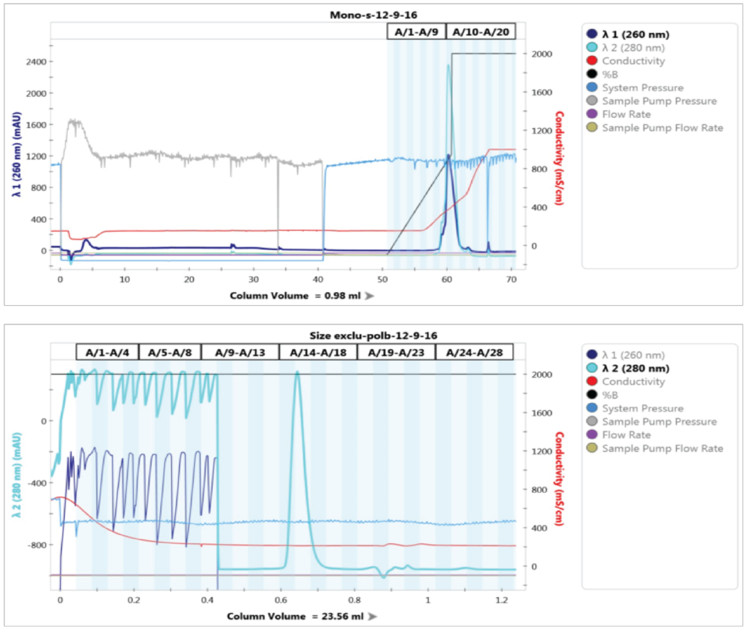

Supplementary Figure S1 (Panels D, E).
(D) The scheme used for recombinant protein expression and purification of the GST tagged and His-tagged proteins, in *E. coli*.
(E) Representative chromatograph of Polβ(WT) purified by Mono-S column and gel filtration column chromatography.

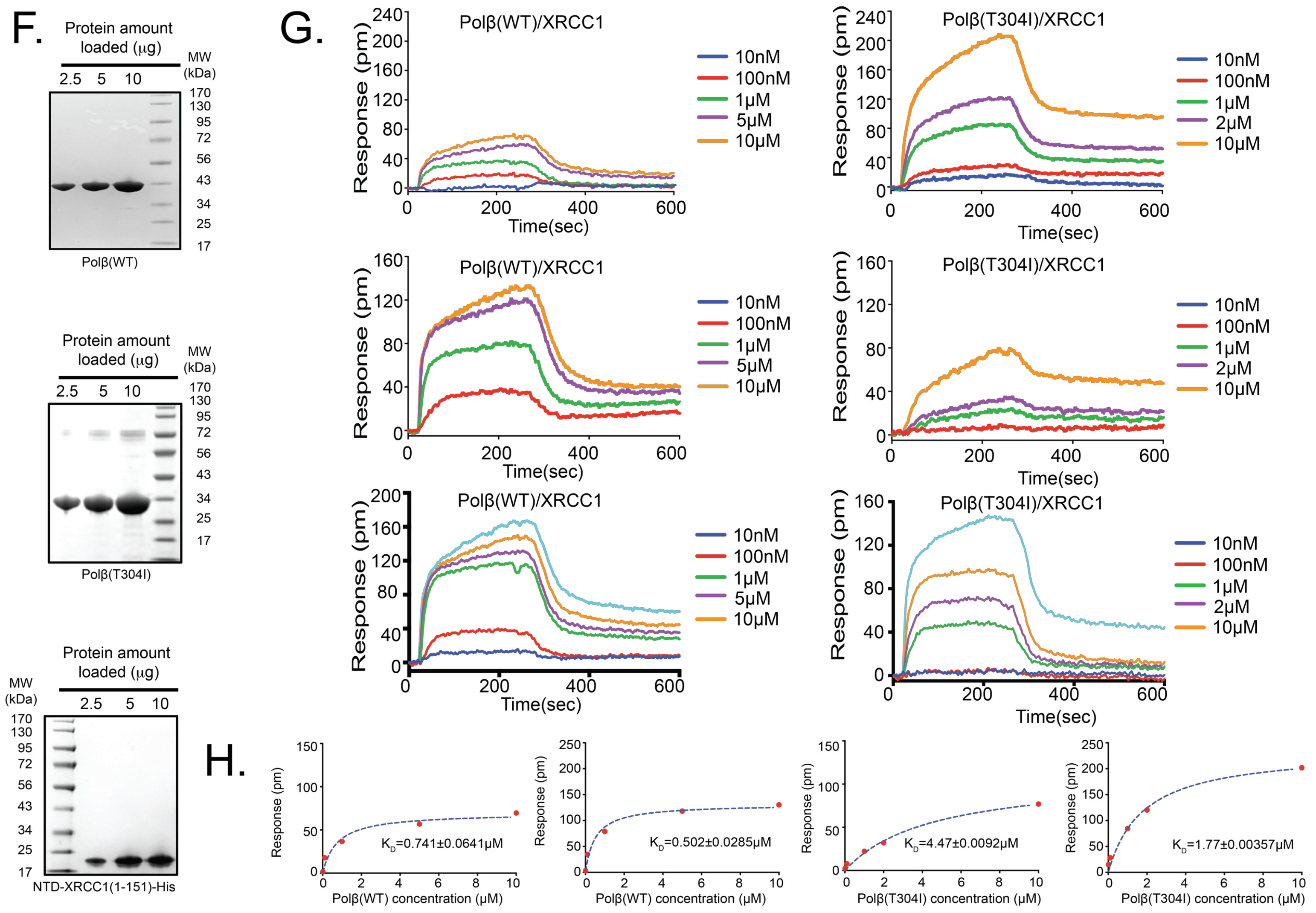

Supplementary Figure S1 (Panels F-H).

(F) Purified proteins examined by Coomassie blue staining following SDS-PAGE gel electrophoresis: Top panel: Polβ(WT), Middle panel: Polβ(T304I) and Bottom panel: NTD-XRCC1(1-151)-His. Molecular weights, as determined by marker proteins, are indicated.

(G) The binding affinity of Polβ(WT)/XRCC1 and Polβ(T304I)/XRCC1 was examined with the OpenSPR assay. All the raw data plots are shown here.

(H) Binding affinity of Polβ(WT) with the N-terminal fragment of XRCC1 (NTD-XRCC1, residues 1-151) and of Polβ(T304I) with NTD-XRCC1 was examined with the OpenSPR assay. The data analyzed from two independent experiments are shown here.

I.

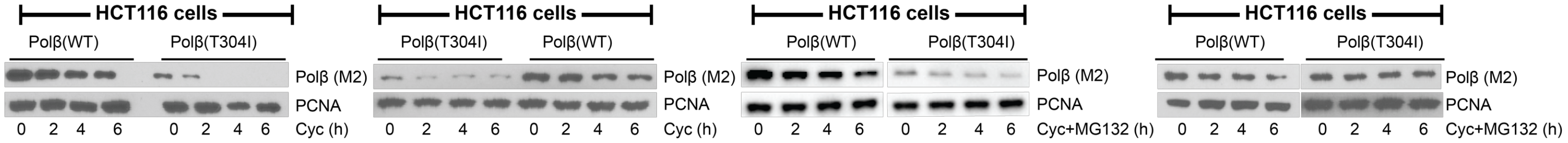

J.

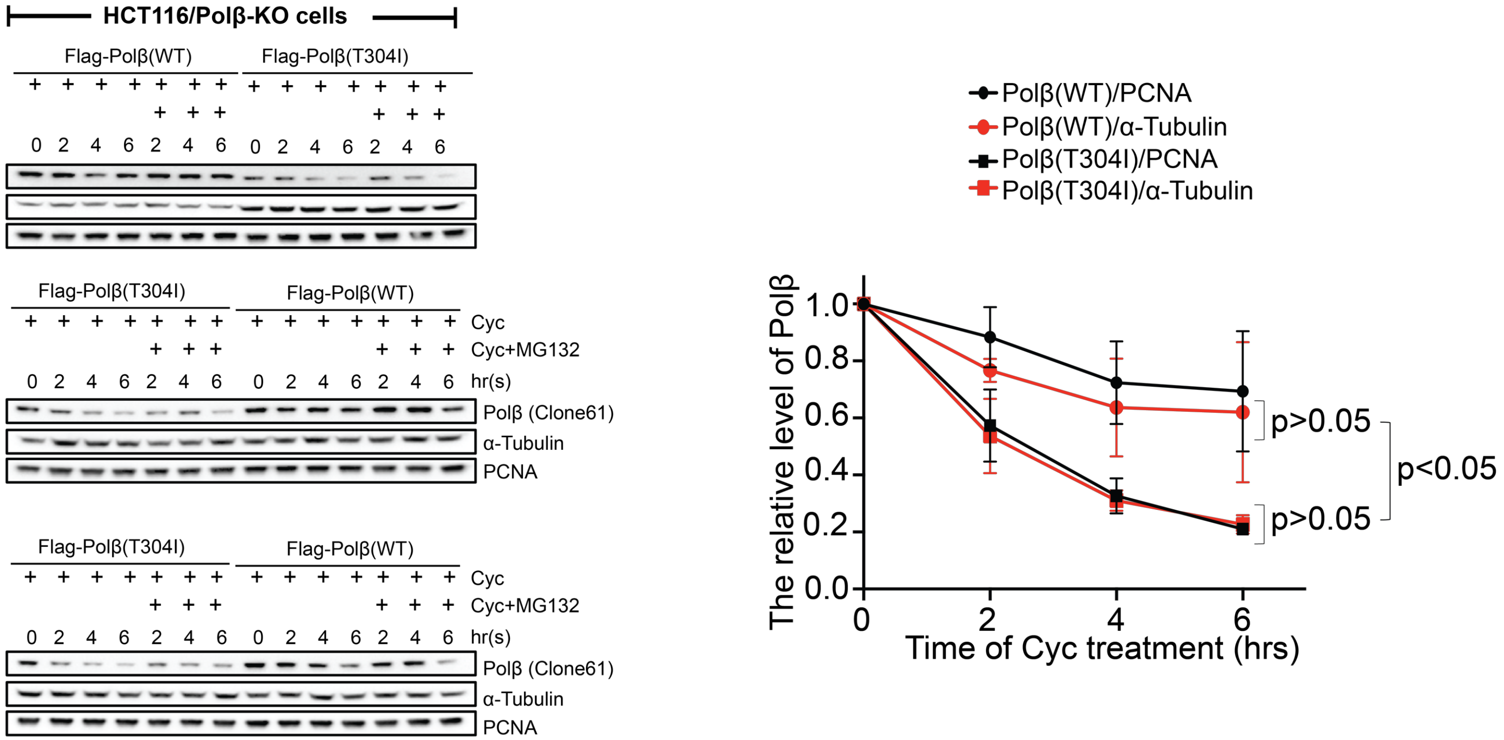

Supplementary Figure S1 (Panels I-J).

(I) Cycloheximide (Cyc) treatment of HCT116 cells results in the enhanced degradation of Polβ(T304I) as compared to Polβ(WT). MG132 treatment stabilizes the degradation of Polβ(T304I) induced by Cyc. The images of two replicate experiments for **Figure 1D** are shown here. Antibodies used for immunoblot are indicated on the right of each panel. The time (h) of treatment with Cyc or Cyc+MG132 are shown along the bottom of each panel.

(J) Left panels: The Polβ(T304I) mutant protein degrades rapidly when expressed in HCT116/Polβ-KO cells. The immunoblot images of cycloheximide (Cyc) treatment resulting in the degradation of Polβ(T304I) when expressed in HCT116/Polβ-KO cells (three independent experiments) are shown here. The level of Polβ, PCNA and α-tubulin in the immunoblots was quantified using Image Lab (Bio-Rad). Right panel: The relative level of Polβ was calculated by determining the ratio of Polβ to PCNA and of Polβ to α-tubulin. The results are plotted, showing the mean ±SD (three independent experiments). The relative level of Polβ(WT) was compared to Polβ(T304I) in HCT116/Polβ-KO cells treated with Cyc; $p<0.05$, as determined by regular two-way ANOVA; The relative level of Polβ(WT) and Polβ(T304I), calculated using the ratio of Polβ/PCNA was compared to the corresponding protein calculated using the ratio of Polβ/α-tubulin in HCT116/Polβ-KO cells treated with Cyc; $p>0.05$, as determined by regular two-way ANOVA.

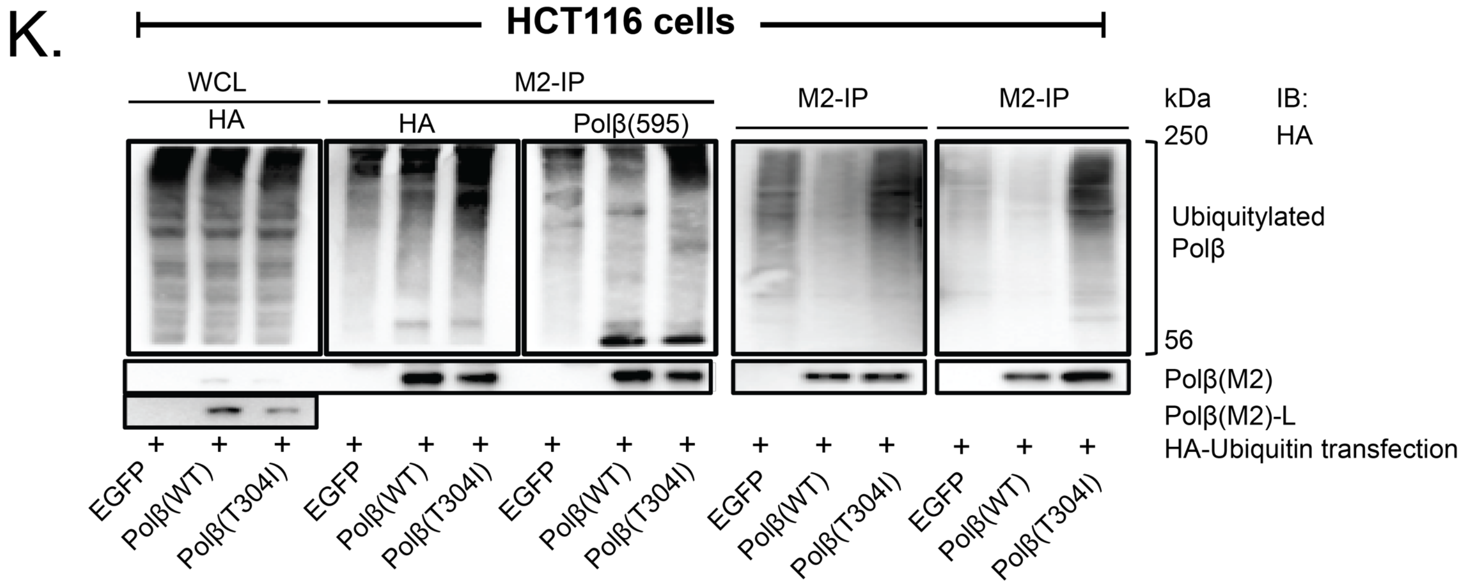

Supplementary Figure S1 (Panel K).
(K) Polβ(T304I) mutation results in enhanced ubiquitylation of Polβ in HCT116 cells. The images of three replicate experiments for **Figure 1E** are shown here. Antibodies used for immunoblot are indicated on the right or on the top of the panel.

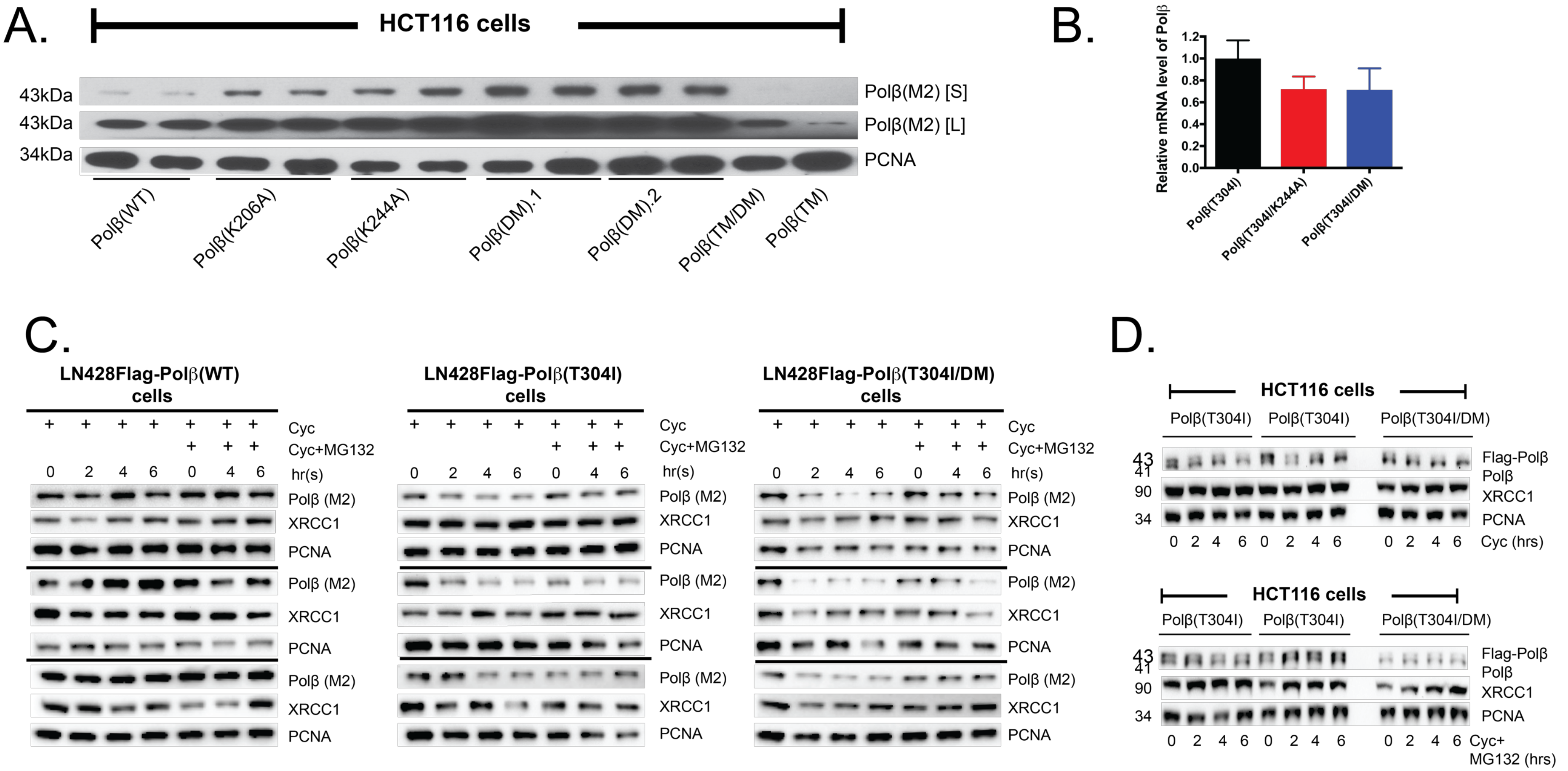

Supplementary Figure S2. Blocking ubiquitylation does not promote the stability of the cancer mutant protein Polβ(T304I).

(A) Immunoblot showing the basal level of Flag-Polβ(WT), Flag-Polβ(K206A), Flag-Polβ(K244A), Flag-Polβ(DM) or Flag-Polβ(TM) expressed in HCT116 cells. The double mutation (K206A/K244A) is denoted as DM and the triple mutation (L301R/V303R/V306R) is denoted as TM. The level of PCNA is shown as a loading control. Molecular weights, as determined by marker proteins, are indicated on the left. Antibodies used for immunoblot are indicated on the right. [S] = short exposure time; [L] = long exposure time.

(B) The relative mRNA level of Polβ in HCT116 cells expressing Flag-Polβ(T304I), Flag-Polβ(T304I/K244A) or Flag-Polβ(T304I/DM). Comparison by ordinary one-way ANOVA; not significant.

(C) Immunoblot showing the level of XRCC1 and Flag-Polβ(WT), Flag-Polβ(T304I) or Flag-Polβ(T304I/DM) in the indicated LN428 cell lines treated with cycloheximide (Cyc) or Cyc + MG132. The level of PCNA is shown as a loading control. Images of three independent experiments for **Figure 2E** are shown.

(D) Immunoblot showing the level of XRCC1, Flag-Polβ(T304I) or Flag-Polβ(T304I/DM) in the indicated HCT116 cells treated with cycloheximide (Cyc) or Cyc + MG132. The level of PCNA is shown as a loading control.

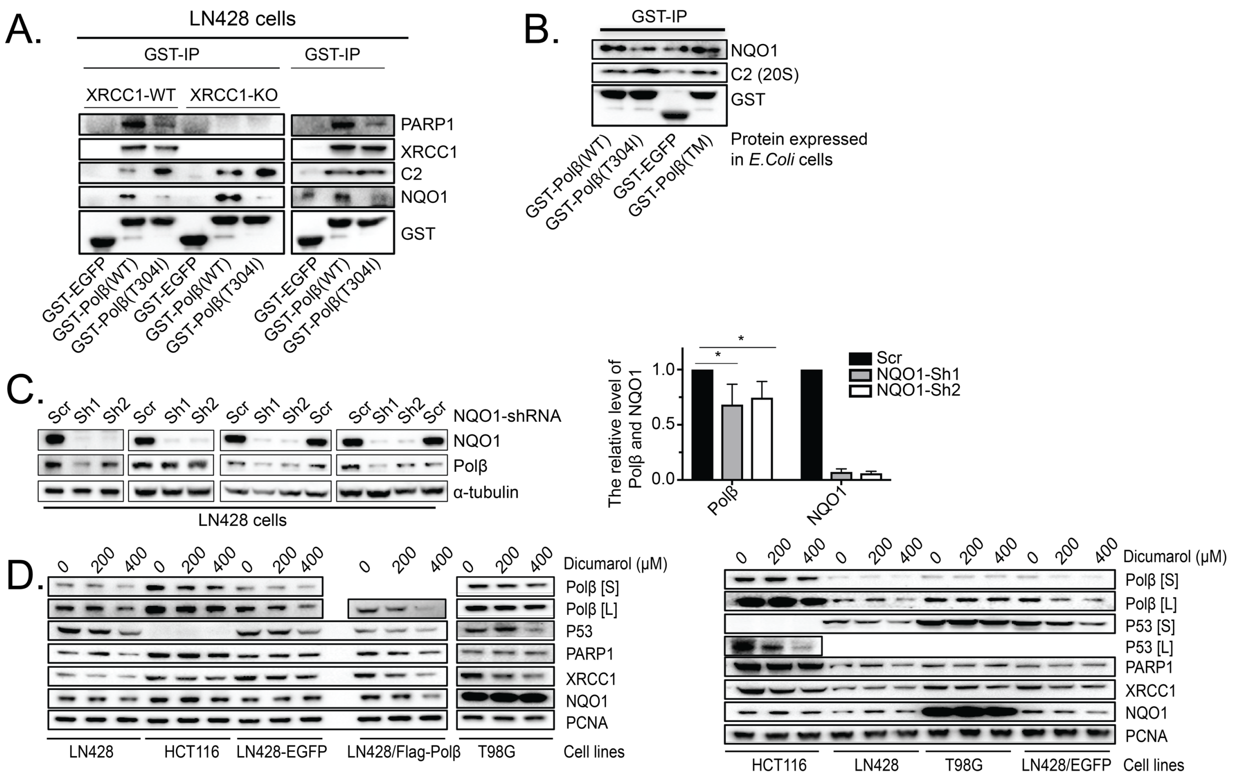

Supplementary Figure S3 (Panels A-D).
(A) Glutathione-agarose/GST-Polβ(WT) (or Glutathione-agarose bound to GST-Polβ mutants or GST-EGFP, as indicated) was incubated with cell lysates prepared from LN428 cells with or without XRCC1-KO. The level of bound NQO1, PARP1, XRCC1, or C2 was examined by immunoblotting and shown. The images shown are the two additional independent experiments described in **Figure 3C**.
(B) The cancer mutant Polβ(T304I) has reduced binding to NQO1 as compared to Polβ(WT). Glutathione-agarose was mixed with cell lysates prepared from BL21-Codonplus-RP cells expressing GST-Polβ(WT), GST-Polβ(T304I), GST-EGFP or GST-Polβ(TM) to bind the indicated GST tagged proteins. Glutathione-agarose/GST-Polβ(WT) (or Glutathione-agarose bound to GST-Polβ mutants or GST-EGFP, as indicated) was incubated with cell lysates prepared from T98G cells. Shown is an immunoblot indicating the level of bound NQO1 or C2. Antibodies used for immunoblot are indicated on the right.
(C) NQO1 knockdown results in low basal level of Polβ in LN428 cells. LN428 cells was transduced by lentiviral vectors expressing scramble (Scr) shRNA or NQO1 short hairpin RNA (shRNA) to knockdown NQO1. Stable cell lines were established. WCL (25μg) was loaded in each lane and the level of NQO1, Polβ and α-tubulin was examined by immunoblotting. The images of four independent experiments are shown. The relative level of Polβ was quantified using Image Lab software (Bio-Rad) and the relative level of Polβ and NQO1 was calculated by the ratio of band densitometry of Polβ/α-tubulin and NQO1/α-tubulin and plotted as shown. Results are the mean ±SD of four independent experiments; *$p<0.05$, compared to cells expressing SCR-shRNA with t-test.
(D) Dicumarol treatment induces the degradation of endogenous Polβ and p53 (positive control) in human cells. The level of Polβ, p53, PARP1, XRCC1, NQO1 and PCNA in WCL was determined by immunoblot analysis of cell lysates prepared from control cells or after treatment with dicumarol (0, 200μM or 400μM) for 5 hrs (the cell lines as indicated). The images shown are the two additional independent experiments described in **Figure 3E**. Antibodies used for immunoblot are indicated on the right of each panel. [S] = short exposure time; [L] = long exposure time.

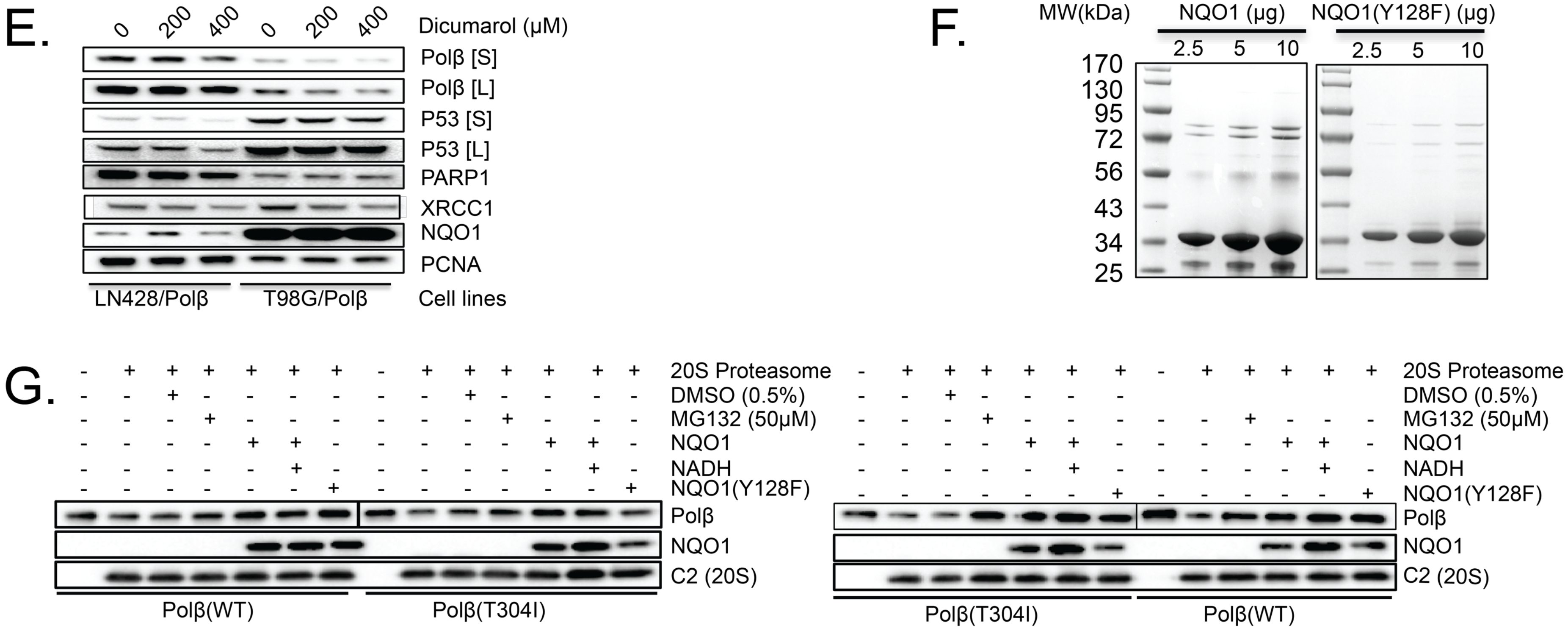

Supplementary Figure S3 (Panels E-G).

(E) Dicumarol treatment induces the degradation of Flag-Polβ(WT) when expressed in LN428 or T98G cells. The level of Flag-Polβ, p53, PARP1, XRCC1, NQO1 and PCNA in WCL was determined by immunoblot analysis of cell lysates prepared from control cells or after treatment with dicumarol (0, 200μM or 400μM) for 5 hrs (the cell lines as indicated). Antibodies used for immunoblot are indicated on the right. [S] = short exposure time; [L] = long exposure time.

(F) Purified NQO1 and NQO1(Y128F) was examined by Coomassie blue staining following SDS-PAGE gel electrophoresis. Molecular weights, as determined by marker proteins, are indicated on the left.

(G) NQO1 and MG132 protects Polβ from 20S proteasome-mediated degradation *in vitro*. Purified Polβ(WT) or Polβ(T304I) (200ng) was incubated with 20S proteasome alone or in the presence of DMSO (0.5%), MG132 (50μM), NQO1 (500ng), NQO1(Y128F) (500ng) or NQO1 (500ng) plus 5mM NADH, at 37˚C for 1 hr. The images shown are the additional two independent experiments described in **Figure 3F**.

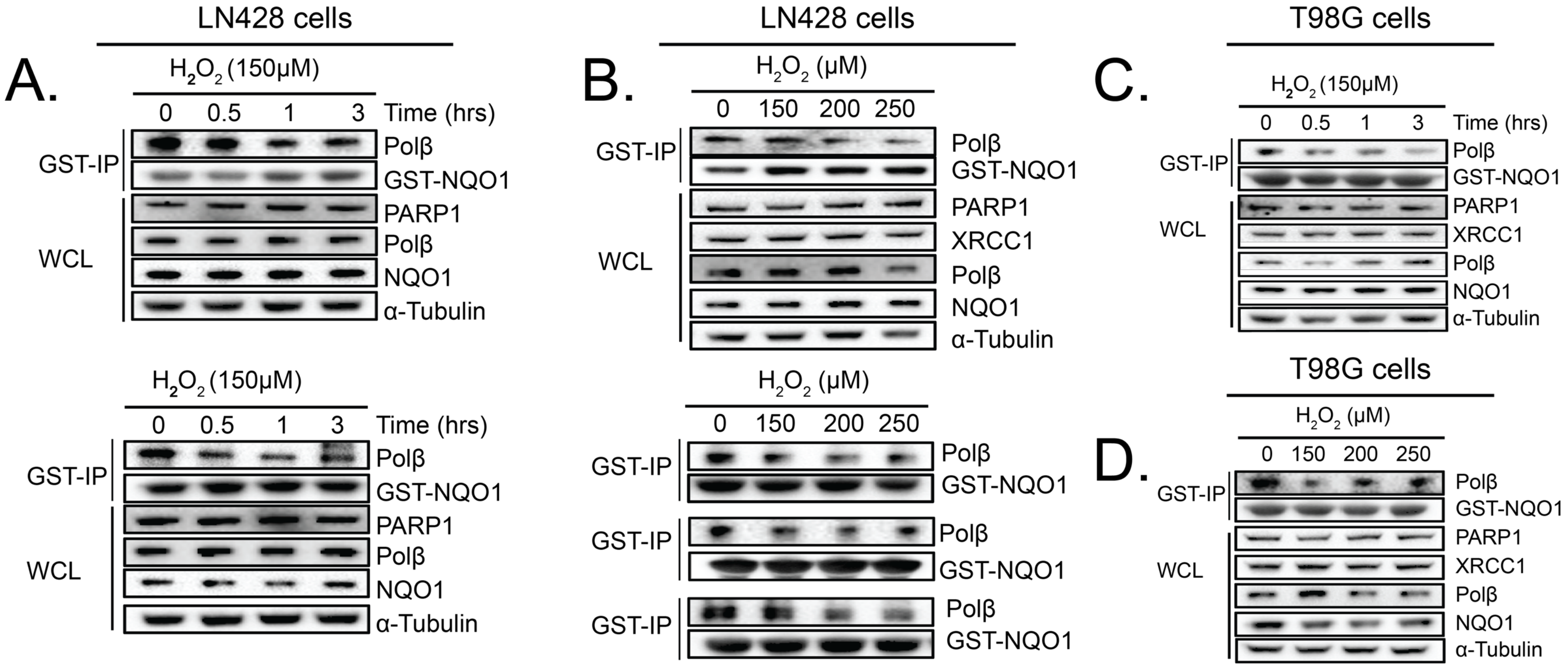

Supplementary Figure S4 (Panels A-D). Oxidative stress promotes the dissociation of Polβ and NQO1 and enhances the association of Polβ with XRCC1.
(A) H_2O_2 treatment of LN428 cells promotes the dissociation of the Polβ / NQO1 complex. Two replicate immunoblots (see **Figure 4A**) show the proteins in LN428 cell lysates bound to GST-NQO1, including Polβ, PARP1 and XRCC1; respectively, and the variation of the bound proteins upon treatment of LN428 cells with H_2O_2 (0-3 hrs, 150mM). Antibodies used for immunoblot are indicated on the right of each panel.
(B) H_2O_2 treatment of LN428 cells (dose response) promotes the dissociation of the Polβ / NQO1 complex. Four replicate immunoblots (see **Figure 4B**) show the proteins in LN428 cell lysates bound to GST-NQO1, including Polβ, PARP1 and XRCC1, respectively, and the variation of the bound proteins upon treatment of LN428 cells with H_2O_2 (3 hrs, 0-250μM). Antibodies used for immunoblot are indicated on the right of each panel.
(C) H_2O_2 treatment of T98G cells promotes the dissociation of the Polβ / NQO1 complex – a time course. The immunoblot shows the proteins in T98G cell lysates bound to GST-NQO1, including Polβ, PARP1 and XRCC1; respectively, and the variation of the bound proteins upon treatment of T98G cells with H_2O_2 (0-3 hrs, 150μM). Antibodies used for immunoblot are indicated on the right.
(D) H_2O_2 treatment of T98G cells (dose response) promotes the dissociation of the Polβ / NQO1 complex. The immunoblot shows the proteins in T98G cell lysates bound to GST-NQO1, including Polβ, PARP1 and XRCC1; respectively, and the variation of the bound proteins upon treatment of T98G cells with H_2O_2 (3 hrs, 0-250μM). Antibodies used for immunoblot are indicated on the right.

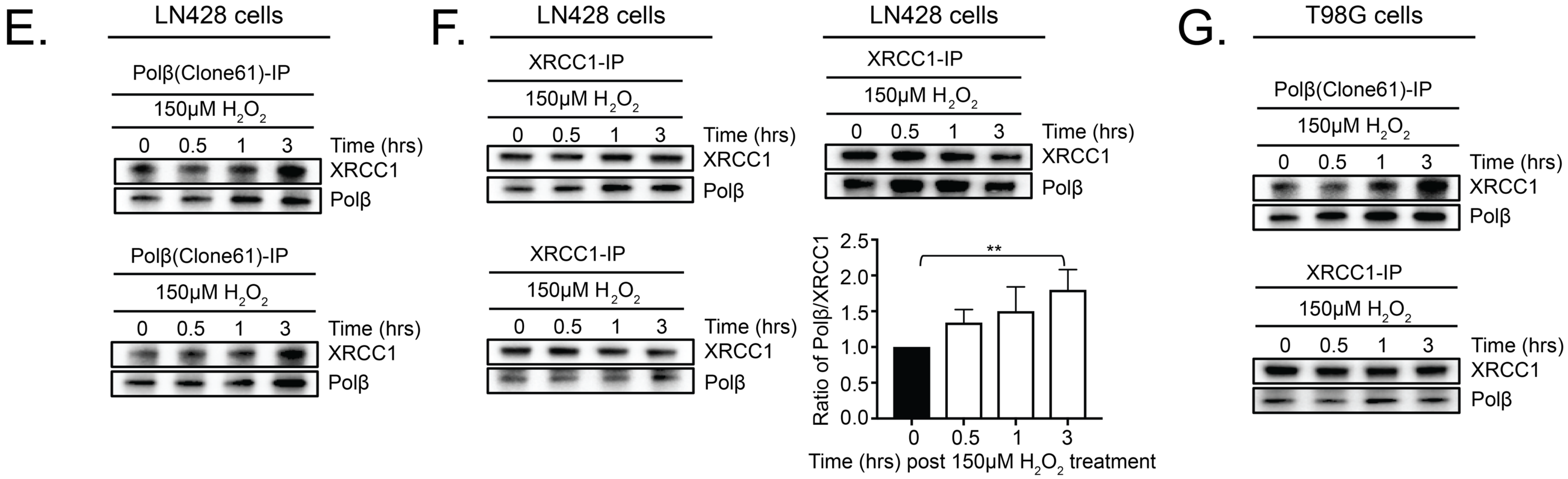

Supplementary Figure S4 (Panels E-G). Oxidative stress promotes the dissociation of Polβ and NQO1 and enhances the association of Polβ with XRCC1.
(E) H_2O_2 treatment promotes the association of Polβ with XRCC1 in LN428 cells. Two replicate immunoblots (see **Figure 4D**) show the proteins immunoprecipitated using an Ab to Polβ (monoclonal Ab, clone 61). Proteins were analyzed from control LN428 cells or those treated with H_2O_2 (150μM, 0.5-3 hrs). The immunoprecipitated proteins were probed by immunoblot for the level of Polβ and XRCC1. Antibodies used for immunoblot are indicated on the right of each panel.
(F) H_2O_2 treatment promotes the association of XRCC1 with Polβ in LN428 cells. Three replicate immunoblots show the proteins immunoprecipitated using an Ab to XRCC1. Proteins were analyzed from control LN428 cells or those treated with H_2O_2 (150μM, 0.5-3 hrs). The immunoprecipitated proteins were probed by immunoblot for the level of Polβ and XRCC1. The ratio of XRCC1/Polβ was quantified and plotted; * $p<0.05$, compared to time=0. One-way ANOVA with Dunnett's multiple comparisons test was used for the plot (bottom right panel).
(G) H_2O_2 treatment promotes the association of Polβ with XRCC1 in T98G cells. Top panel: The representative immunoblot shows the proteins immunoprecipitated using an Ab to Polβ (monoclonal Ab, clone 61). Proteins were analyzed from control T98G cells or those treated with H_2O_2 (150μM, 0.5-3 hrs). The immunoprecipitated proteins were probed by immunoblot for the level of Polβ and XRCC1. Bottom panel: The representative immunoblot shows the proteins immunoprecipitated using an Ab to XRCC1. Proteins were analyzed from control T98G cells or those treated with H_2O_2 (150μM, 0.5-3 hrs). The immunoprecipitated proteins were probed by immunoblot for the level of Polβ and XRCC1. Antibodies used for immunoblot are indicated on the right of each panel.

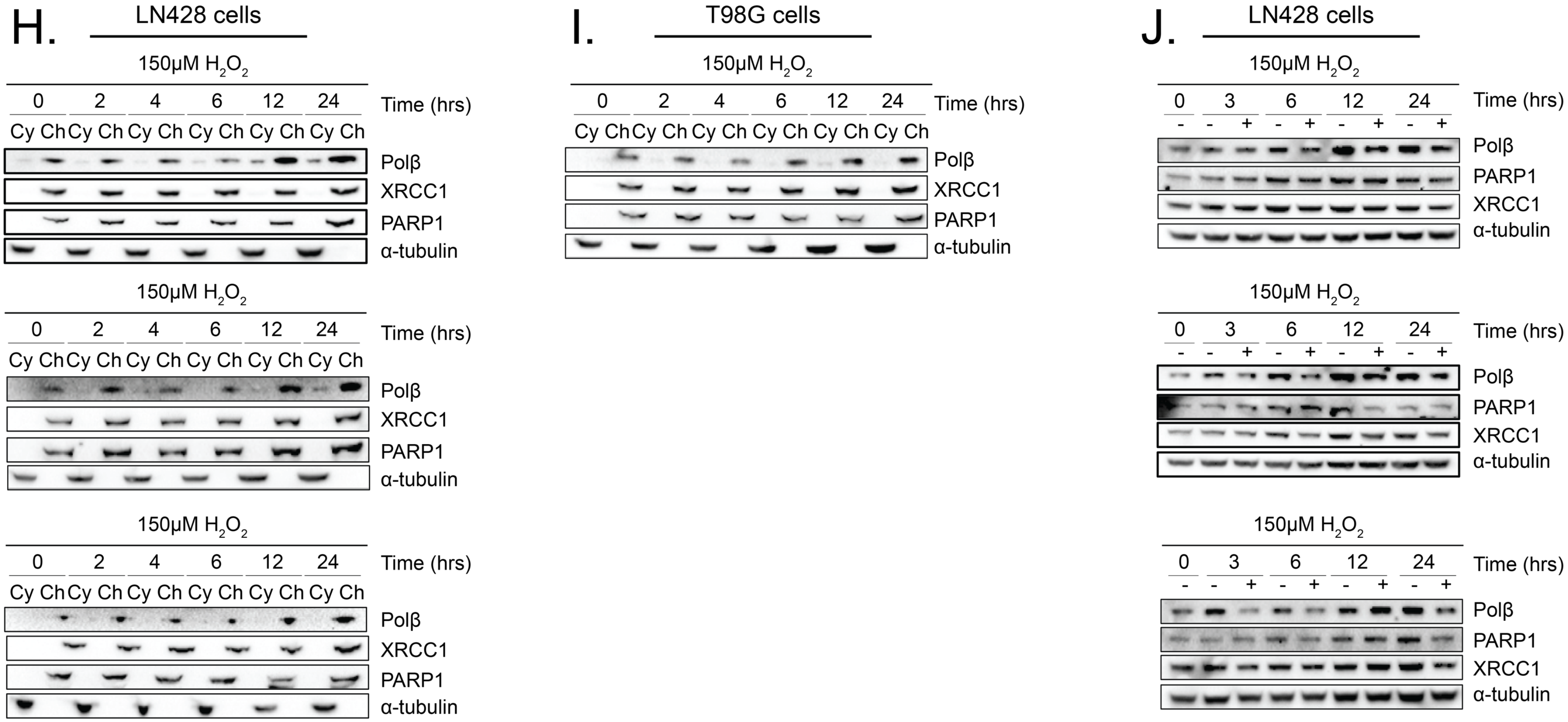

Supplementary Figure S4 (Panels H-J). Oxidative stress promotes the dissociation of Polβ and NQO1 and enhances the association of Polβ with XRCC1. **(H)** Oxidative stress impacts the subcellular distribution of Polβ but not XRCC1 in LN428 cells. Three replicate immunoblots (see **Figure 4F**) show the subcellular distribution of Polβ in LN428 cells: control cells or those treated with H_2O_2 (150μM, 0-24 hrs). Proteins from the cytosolic fraction (Cy) or the chromatin fraction (Ch) from LN428 cells were isolated and the level of Polβ, PARP1, XRCC1 and α-tubulin was examined by immunoblot. Antibodies used for immunoblot are indicated on the right.
(I) Oxidative stress impacts the subcellular distribution of Polβ in T98G cells. The subcellular distribution of Polβ in T98G cells: control cells or those treated with H_2O_2 (150μM, 0-24 hrs). Proteins from the cytosolic fraction (Cy) or the chromatin fraction (Ch) from LN428 cells were isolated and the level of Polβ, PARP1, XRCC1 and α-tubulin was examined by immunoblot. Antibodies used for immunoblot are indicated on the right.
(J) Oxidative stress does not induce increased overall levels of Polβ in LN428 cells. Three replicate immunoblots (see **Figure 4F**) show the level of Polβ in LN428 with H_2O_2 treatment. Whole cell lysates (WCL) of LN428 cells (control cells or those treated with H_2O_2, 150μM, 0-24 hrs) were evaluated by immunoblot and the level of Polβ, PARP1, XRCC1 and α-tubulin was examined. Antibodies used for immunoblot are indicated on the right.

K.

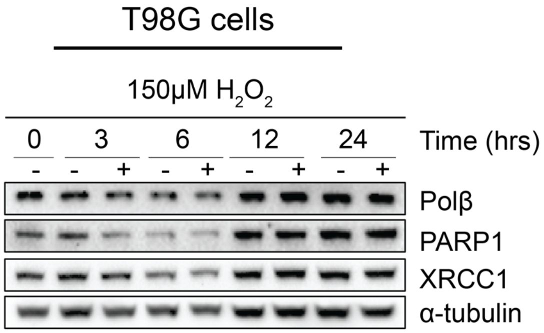

L.

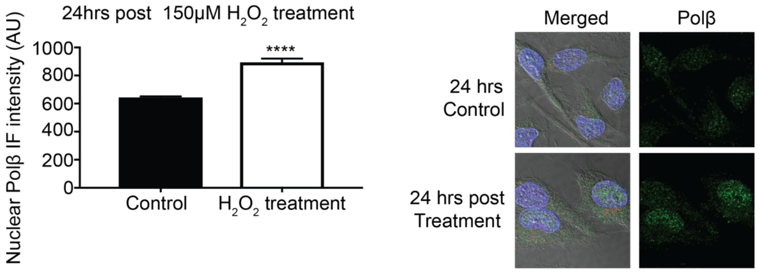

Supplementary Figure S4 (Panels K-L). Oxidative stress promotes the dissociation of Polβ and NQO1 and enhances the association of Polβ with XRCC1.

(K) Oxidative stress does not induce increased overall levels of Polβ inT98G cells. Whole cell lysates (WCL) of T98G cells (control cells or those treated with H_2O_2; 150μM, 0-24 hrs) were evaluated by immunoblot and the level of Polβ, PARP1, XRCC1 and α-tubulin was examined. Antibodies used for immunoblot are indicated on the right.

(L) Oxidative stress promotes nuclear accumulation of Polβ. LN428 cells were treated with H_2O_2 (150μM, 24 hrs) and fixed. Polβ was probed with anti-Polβ antibody, and the nuclear compartment was defined by staining with DAPI. Confocal images were collected and nuclear staining intensity was quantified using a custom analysis macro written for NIS-Elements. Quantified data was compared using Student's T-test. Analysis represents 100-200 cells for each condition; ****p<0.00001.

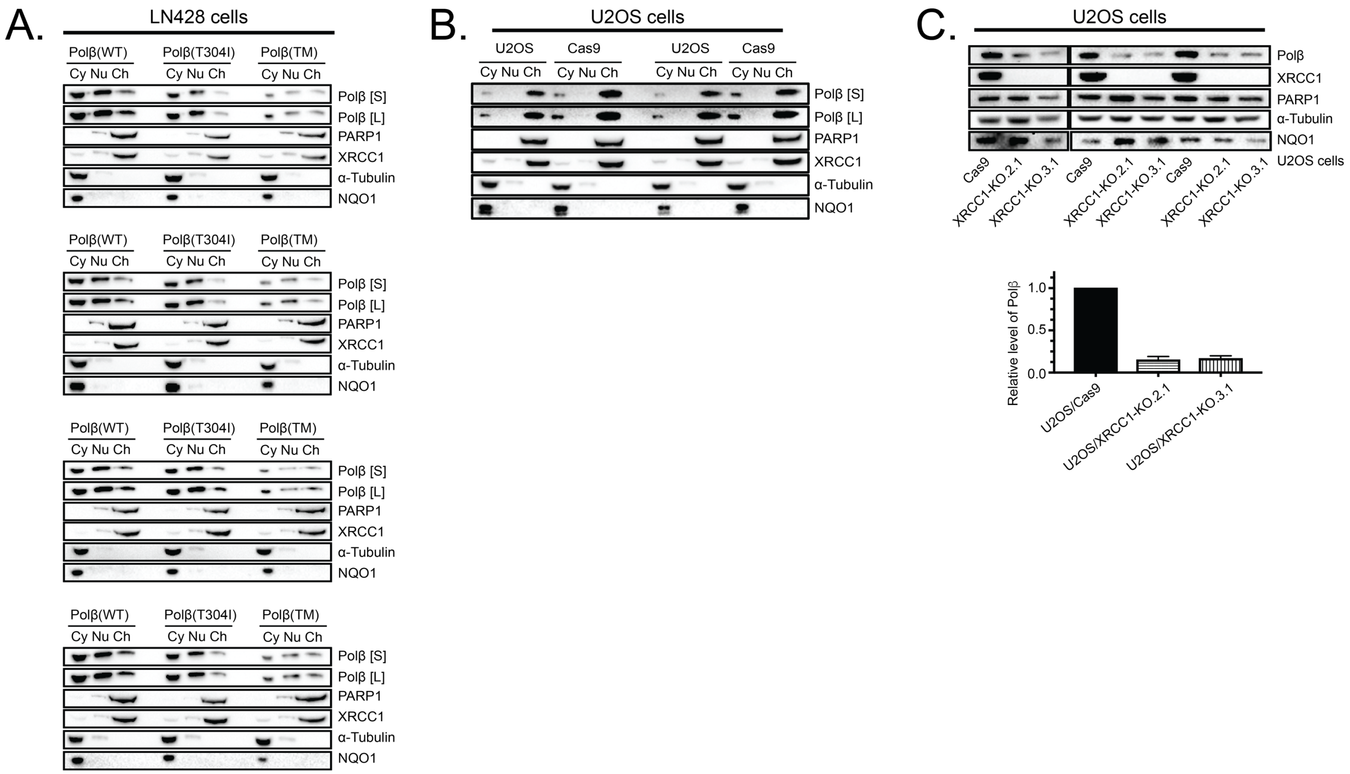

Supplementary Figure S5 (Panels A-C). Loss of XRCC1 expression impacts Polβ stability and chromatin localization of Polβ.

(A) Polβ levels are reduced in the chromatin fraction when unable to bind to XRCC1. Proteins of the cytosolic fraction, soluble nuclear fraction and the chromatin fraction from LN428 cells expressing Flag-Polβ(WT), Flag-Polβ(T304I) or Flag-Polβ(TM) were isolated and the level of Polβ, PARP1, XRCC1, α-tubulin and NQO1 was examined by immunoblot. The images shown are four independent immunoblot experiments related to **Figure 5A**. Antibodies used for immunoblot are indicated; [S] = short exposure time; [L] = long exposure time.

(B) The subcellular distribution of Polβ in U2OS cells in the presence or absence of Cas9. Proteins of the cytosolic fraction, soluble nuclear fraction and the chromatin fraction from U2OS (control) and U2OS/Cas9 cells were isolated and the level of Polβ, PARP1, XRCC1, α-tubulin and NQO1 was examined by immunoblot. The images of two independent experiments was shown here. Antibodies used for immunoblot are indicated; [S] = short exposure time; [L] = long exposure time.

(C) The basal protein expression level of Polβ in U2OS cells in the presence or absence of XRCC1. Top panel: WCL from U2OS/Cas9 (control) and U2OS/XRCC1-KO cells (clones 2.1 and 3.1) were isolated and the level of Polβ, PARP1, XRCC1, α-tubulin and NQO1 was examined by immunoblot. The images of three independent immunoblot experiments are shown. Antibodies used for immunoblot are indicated. Bottom panel: The level of Polβ was evaluated by densitometry and the relative level of Polβ in the fractions was quantified. The ratio of Polβ/α-tubulin was calculated and plotted as shown. Results indicate the mean ± SD of three independent experiments.

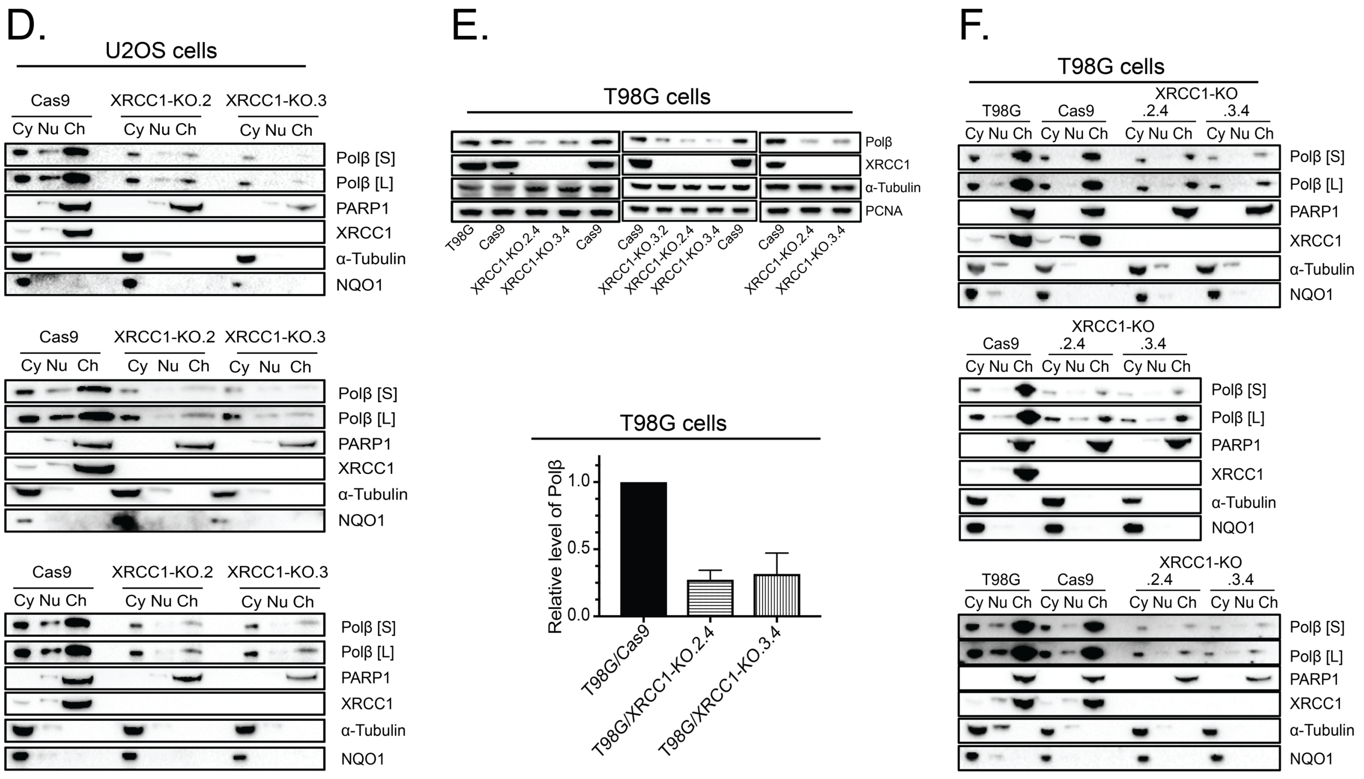

Supplementary Figure S5 (Panels D-F). Loss of XRCC1 expression impacts Polβ stability and chromatin localization of Polβ.

(D) The subcellular distribution of Polβ in U2OS cells in the presence or absence of XRCC1. Proteins of the cytosolic fraction, soluble nuclear fraction and the chromatin fraction from U2OS/Cas9 (control) and U2OS/XRCC1-KO cells (clones 2.1 and 3.1) were isolated and the level of Polβ, PARP1, XRCC1, α-tubulin and NQO1 was examined by immunoblot. The images of three independent immunoblot experiments are shown, in support of **Figure 5B**. Antibodies used for immunoblot are indicated; [S] = short exposure time; [L] = long exposure time.

(E) The basal protein expression level of Polβ in T98G cells in the presence or absence of XRCC1. Top panel: WCL from T98G/Cas9 (control) and T98G/XRCC1-KO cells (clones 2.4 and 3.4) were isolated and the level of Polβ, PARP1, XRCC1 and α-tubulin was examined by immunoblot. Antibodies used for immunoblot are indicated. The images of three independent immunoblot experiments are shown. Bottom panel: The level of Polβ was evaluated by densitometry and the relative level of Polβ in the fractions was quantified. The ratio of Polβ/α-tubulin was calculated and plotted as shown. Results indicate the mean ± SD for three independent experiments.

(F) The subcellular distribution of Polβ in T98G cells in the presence or absence of XRCC1. Proteins of the cytosolic fraction, soluble nuclear fraction and the chromatin fraction from T98G/Cas9 (control) and T98G/XRCC1-KO cells (clones 2.4 and 3.4) were isolated and the level of Polβ, PARP1, XRCC1, α-tubulin and NQO1 was examined by immunoblot. The images of three independent immunoblot experiments are shown, in support of **Figure 5C**. Antibodies used for immunoblot are indicated; [S] = short exposure time; [L] = long exposure time.

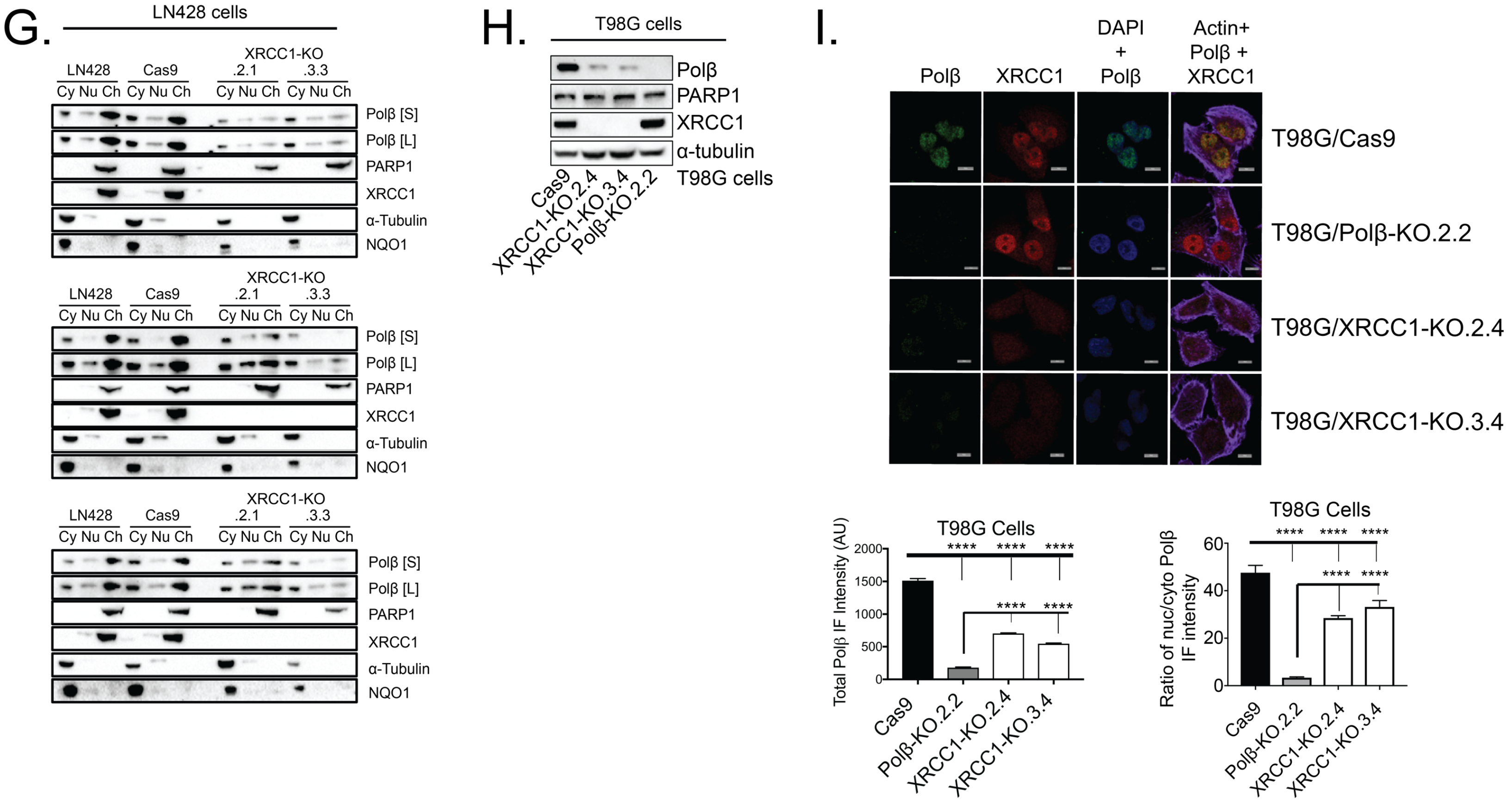

Supplementary Figure S5 (Panels G-I). Loss of XRCC1 expression impacts Polβ stability and chromatin localization of Polβ.

(G) The subcellular distribution of Polβ in LN428 cells in the presence or absence of XRCC1. Protein fractions of the cytosol, nucleus and chromatin from LN428/Cas9 (control) and LN428/XRCC1-KO cells (clones 2.1 and 3.3) were isolated and the level of Polβ, PARP1, XRCC1, α-tubulin and NQO1 was examined by immunoblot. The images of three independent immunoblot experiments are shown. Antibodies used for immunoblot are indicated; [S] = short exposure time; [L] = long exposure time.

(H) Immunoblotting assay to confirm the loss of XRCC1 in the T98G/XRCC1-KO cells and the loss of Polβ in the T98G/Polβ-KO cells.

(I) Top panel: The subcellular distribution of Polβ and XRCC1 in T98G cells in the presence or absence of XRCC1 was evaluated by immunofluorescence confocal microscopy. Polβ and XRCC1 were probed with anti-Polβ and anti-XRCC1 antibodies, and the nuclear and cytoplasmic compartments were defined by staining with DAPI and phalloidin conjugated to AlexFluor 647, respectively. Images were collected and staining intensity was quantified using a custom analysis macro written for NIS-Elements. Quantified data was compared using one-way ANOVA followed by Tukey's multiple comparison test. T98G cells in which XRCC1 was knocked out show a reduction in both overall levels of Polβ (bottom left panel) and nuclear/cytoplasmic ratios of Polβ (bottom right panel). Representative images are shown, 100-200 cells were analyzed per condition; ****$p<0.0001$.

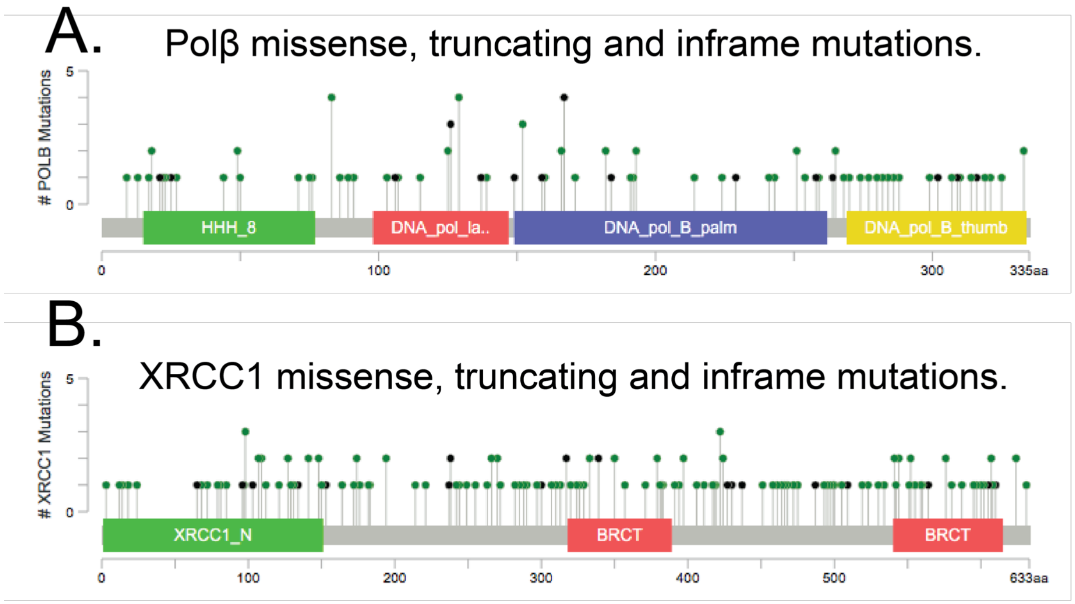

Supplementary Figure S6. Mutations in the POLB and XRCC1 genes identified in human cancers.
(A) Missense, truncating and in-frame mutations of the POLB gene by querying 48077 samples in 177 studies via the cBioportal analysis tool (1,2).
(B) Missense, truncating and in-frame mutations of the XRCC1 gene by querying 48077 samples in 177 studies via the cBioportal analysis tool (1,2).
Key: Missense mutations shown in green; truncating mutations shown in black and In-frame mutations shown in brown.

References Cited

1. Gao, J., Aksoy, B.A., Dogrusoz, U., Dresdner, G., Gross, B., Sumer, S.O., Sun, Y., Jacobsen, A., Sinha, R., Larsson, E. *et al.* (2013) Integrative analysis of complex cancer genomics and clinical profiles using the cBioPortal. *Sci Signal*, **6**, pl1.
2. Cerami, E., Gao, J., Dogrusoz, U., Gross, B.E., Sumer, S.O., Aksoy, B.A., Jacobsen, A., Byrne, C.J., Heuer, M.L., Larsson, E. *et al.* (2012) The cBio cancer genomics portal: an open platform for exploring multidimensional cancer genomics data. *Cancer discovery*, **2**, 401-404.
